# The SIRT1 N‐Terminal Domain as a Common Binding Interface for PPARγ Anchoring

**DOI:** 10.1002/prot.70022

**Published:** 2025-07-17

**Authors:** Caique Camargo Malospirito, Gabriel Ernesto Jara, Víctor Ulian Antunes, Giovanna Blazutti Elias, Marieli Mariano Goncalves Dias, Fernanda Aparecida Heleno Batista, Paulo Sergio Lopes de Oliveira, Ana Carolina Migliorini Figueira

**Affiliations:** ^1^ Brazilian Biosciences National Laboratory (LNBio) Brazilian Center for Research in Energy and Materials (CNPEM) Campinas Brazil; ^2^ Graduate Program in Molecular and Morpho Functional Biology Institute of Biology, State University of Campinas (Unicamp) Campinas Brazil; ^3^ Graduate Program in Pharmaceutical Sciences, Faculty of Pharmaceutic Sciences State University of Campinas (Unicamp) Campinas Brazil; ^4^ Molecular Investigation Laboratory in Cardiology Institute Dante Pazzanese of Cardiology São Paulo Brazil

**Keywords:** insulin resistance, molecular modeling, PPARγ, protein biochemistry, protein biophysics, SIRT1

## Abstract

Insulin resistance, a global health threat linked to type 2 diabetes and obesity, can be addressed by modulating the activity of the Sirtuin 1 (SIRT1), a deacetylase that enhances insulin sensitivity by deacetylating the Peroxisome Proliferator‐Activated Receptor Gamma (PPARγ) at lysine 268 and 293. Understanding the binding interfaces between SIRT1 and PPARγ is critical to developing new strategies to combat insulin resistance. In this study, we present four experimentally supported binding models of SIRT1 with acetylated PPARγ: one at position 268 and three at position 293 (SIRT1‐_K268_PPARγ and SIRT1‐_K293_PPARγ_1‐3_ models). These models were generated through an integration of in silico modeling and in vitro binding affinity assays. Our models revealed that the SIRT1:PPARγ binding interface is structured by SIRT1's 3‐helix bundle in N‐terminus domain (NTD(3HB)) and the catalytic domain (CD). The CD accommodated the acetylated peptide in its active site, while NTD(3HB) anchors PPARγ at a region between loops α1‐β1 and α2′‐α3 within PPARγ's ligand binding domain (LBD). Notably, the SIRT1‐NTD(3HB) consistently bound to the same region of PPARγ in both models, highlighting a common mode for interaction. Through molecular dynamic simulation and binding assays, we demonstrated that either removal of SIRT1‐NTD(3HB) or mutation within PPARγ‐LBD significantly reduces binding affinity, underscoring the role of NTD(3HB) in substrate anchoring. Additionally, we provided evidence of SIRT1 dimerization, with substrate binding inducing its dissociation to form a heterodimer with PPARγ. These findings underscore the importance of the SIRT1 NTD(3HB) in PPARγ anchoring and offer insights into the activation mechanism of SIRT1, with potential implications for drug development targeting insulin resistance.

## 1 Introduction

Metabolic syndrome has evolved into a pressing global health concern. One of its hallmark features is insulin resistance, the body's failure to respond to the hormone insulin. This condition increases the risk of chronic diseases such as type 2 diabetes, leading to a growing demand for medical care and placing a substantial strain on healthcare systems worldwide [1]. In this scenario, the Peroxisome Proliferator‐Activated Receptor Gamma (PPARγ) emerged as a target for antidiabetic drugs due to its role in insulin sensitization. PPARγ is a nuclear receptor that modulates the expression of genes related to insulin sensitivity. One mechanism of promoting insulin sensitization involves a reduction of acetylation levels of PPARγ in adipocytes [2, 3, 4]. The decrease in PPARγ acetylation promotes insulin‐sensitivity outcomes and opens new possibilities for drug discovery targeting PPARγ modulation. The deacetylase Sirtuin 1 (SIRT1) plays an important role in insulin sensitization and PPARγ activation because it catalyzes the removal of the acetyl group from PPARγ at acetyl‐Lysine 268 (K268ac) and 293 (K293ac). As a result of SIRT1 deacetylation, PPARγ acetylation levels decrease, leading to the promotion of the expression of insulin‐sensitivity genes under PPARγ control [2, 3]. Even though the biology behind PPARγ and SIRT1 in insulin sensitivity is well established, the structural mechanism governing their interaction remains unclear.

The expression of insulin‐sensitivity‐related genes requires the formation of the heterodimer between PPARγ and the retinoid X receptor (RXR). The heterodimer binds to specific DNA sequences in the promoter regions of target genes, leading to the modulation of gene expression. Additionally, the classic mechanism of PPARγ activation involves its interaction with coregulatory proteins, which can either enhance or repress its transcriptional activity [5]. Structurally, PPARγ binds to the promoter regions through its DNA‐binding domain (DBD), which is connected to the ligand‐binding domain (LBD) by a hinge region. PPARγ also harbors an N‐terminal unstructured region, the transactivation domain (AF1), an important regulatory component for PPARγ function. Not only does AF1 act as a regulatory domain of PPARγ, but LBD does as well. It contains crucial post‐translation modifications affecting PPARγ activity [6, 7, 8, 9] and is a target of several insulin‐sensitizing drugs aimed to activate PPARγ [10]. The ligand Rosiglitazone is a well‐known insulin‐sensitizing drug that targets PPARγ and activates it by inducing conformational changes in the LBD region, mainly through α12, α3, and the β‐sheet region of the LBD [10]. These structural changes in LBD promote the recruitment of coactivator proteins to promoter regions, increasing the expression of PPARγ target genes [5]. Administration of Rosiglitazone also causes the deacetylation of K268ac and K293ac by SIRT1, which partially explains the insulin‐sensitizing effects of Rosiglitazone. Even though Rosiglitazone leads to insulin sensitivity outcomes, its widespread use is associated with numerous side effects beyond its intended benefits. Therefore, targeting PPARγ activation by enhancing SIRT1‐mediated deacetylation of PPARγ presents new possibilities for developing innovative treatment strategies. This approach could potentially overcome the adverse effects associated with current insulin‐sensitizing drugs such as Rosiglitazone.

SIRT1, the mammalian homolog of the yeast silencing information regulator 2 (Sir2) protein, was originally described as a regulator of gene expression and has been implicated in the extension of the lifespan of organisms. Further research revealed that SIRT1 activity is dependent on the cellular levels of the coenzyme NAD^+^, linking its gene regulatory functions to the cellular energy state. This connection positions SIRT1 as a promising target for developing new approaches to treat metabolic disorders, such as insulin resistance [2, 11, 12, 13]. Structurally, SIRT1 possesses a conserved catalytic domain (CD) core, supplemented by a Zn‐binding domain, and an N‐terminal (NTD) and a C‐terminal (CTD) domain [14, 15]. SIRT1 catalysis requires the binding of NAD^+^ to CD and the proper positioning of acetyl‐peptide (ac‐Peptide), closer to the active histidine (His363) in the active site. This reaction results in the deacetylated substrate, 2‐O‐acetyl‐ADP‐ribose, and nicotinamide, which additionally act as an inhibitory component of SIRT1 activity [3, 16, 17]. While CD is primarily responsible for SIRT1 catalysis, SIRT1‐NTD and ‐CTD primarily act as regulatory domains [18, 19, 20, 21, 22]. These domains promote SIRT1 activity through an allosteric mechanism involving specific regions within the NTD and CTD.

Although NTD and CTD are predominantly disordered, each has an ordered region that is crucial for SIRT1 activity [19, 20, 23]. The essential‐for‐SIRT1‐activity region (ESA) is located in the CTD of SIRT1 and plays a crucial role in modulating SIRT1 activity. The ESA peptide binds to the C‐terminal region of the catalytic core of SIRT1, thereby complementing the β‐sheet region in CD and increasing SIRT1 activity. On the other hand, the NTD has an ordered region harboring a 3‐helix bundle (3HB) important for SIRT1 activity and regulatory features [18, 19, 20, 24, 25]. The 3HB is the target of SIRT1 regulator proteins, such as DBC (Deleted in Breast Cancer 1 protein) and the sirtuin‐activating compound (STAC). DBC binds to the disordered regions in the NTD, causing the exposure of the 3HB region to other protein partners and/or ligands that activate or inhibit SIRT1 activity [15, 26]. Similar to DBC, Resveratrol, a natural polyphenolic STAC that improves glucose tolerance and insulin sensitivity, also binds to 3HB, leading to the repositioning of 3HB in closer proximity to CD, thereby modulating SIRT1 enzymatic activity. This repositioning of 3HB closer to the catalytic core and the importance of the ESA peptide for SIRT1 activity underscore the importance of the domain's rearrangements in SIRT1 activation [19, 20].

While the roles of PPARγ and SIRT1 in enhancing insulin sensitivity are well‐established [2, 3, 4], the structural mechanisms underlying their interaction remain unclear. Current structural studies involving SIRT1 and its substrate binding focus on acetyl‐peptides substrate rather than native proteins, thereby hindering the development of new therapeutic strategies targeting binding interfaces between SIRT1 and native proteins as PPARγ [19, 20, 27]. Here, we combine in silico and in vitro studies to investigate the binding of the native substrate PPARγ to SIRT1 by characterizing their binding interfaces. We propose experimental‐based binding models for K268 and K293 residues: SIRT1‐_K268_PPARγ and SIRT1‐_K293_PPARγ_1–3_. Guided by binding assays and stoichiometry studies, these models reveal that PPARγ binds to SIRT1 through a novel interface formed by SIRT1's CD and NTD(3HB) region. Our findings underscore the NTD(3HB) as common SIRT1 binding interface on PPARγ in both models, highlighting key residues crucial for binding through molecular dynamics simulations (MD simulations), further validated through binding assays experiments.

## 2 Material and Methods

### 2.1 Protein Expression and Purification

**FIGURE 1:**
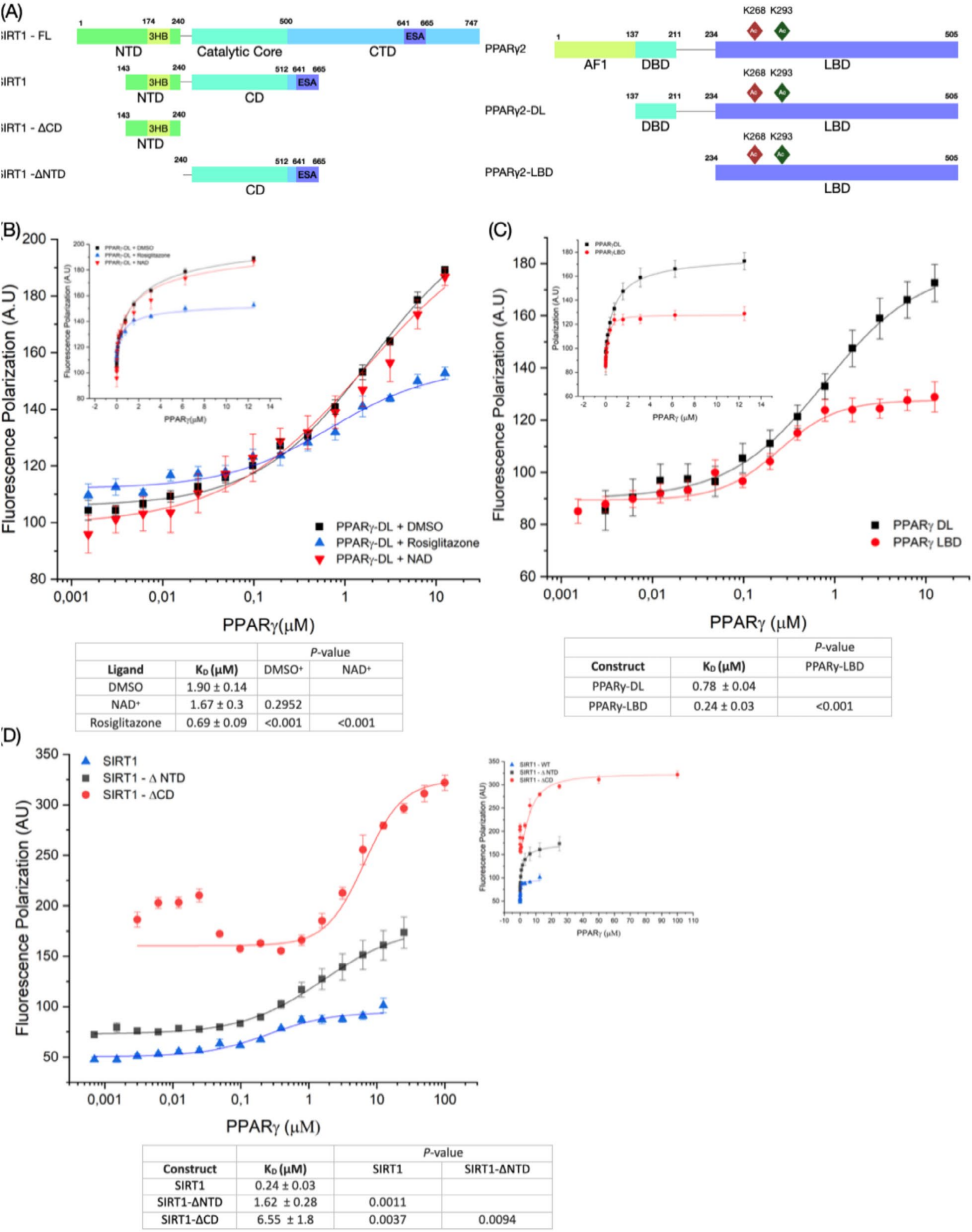
(A) Illustration showing the protein constructs investigated in this study: PPARγ construct (DL and LBD) and SIRT1 constructs (SIRT1, SIRT1‐ΔCD, SIRT1‐ΔNTD). (B) FP curves of PPARγ‐DL in the presence of Rosiglitazone and NAD^+^, measured against labeled‐SIRT1. (C) FP curves comparing different PPARγ constructs binding to labeled‐SIRT1. (D) FP curves for labeled SIRT1, SIRT1‐ΔCD, and SIRT1‐ΔNTD constructs in the presence of increasing concentrations of PPARγ‐LBD. The insert includes a table with the *K*_D_ derived from FP assays, along with the FP curves plotted on a linear scale. The *p* values from the unpaired *t*‐test are also presented in the table.

The expression and purification of PPARγ (isoform 2) constructs (DL and LBD), and mutants (PPARγ K268Q, K293Q, L265R, Q299D, V276R) were performed as described previously [28]. For SIRT1, we took advantage of a soluble construct with an available crystal structure and enzymatically active (Figure 1A, Figure S1) [19]. hSIRT1pET‐SMT3 1‐143CS vector was kindly donated by the National Laboratory of Biomacromolecules, Institute of Biophysics, Chinese Academy of Sciences, and was used to express the construct referred to as SIRT1 in this work. For SIRT1, SIRT1‐ΔNTD, and SIRT1‐ΔCD mutants, the expression and purification were performed following a previously described protocol with the following modification [19]. The immobilized metal affinity chromatography (IMAC) was carried out using Talon Superflow resin (Clontech). The soluble fraction was incubated with it for 1 h at 4°C at a ratio of 1 mL of resin per liter of culture. Then, the mixture was placed in a gravity column (Bio‐Rad) and the resin was washed with 10 column volumes (CV) with wash buffer (20 mM Tris–HCl pH 8.0, 200 mM NaCl, 5% glycerol, and 5 mM imidazole). The tagged protein was eluted using elution buffer (20 mM Tris–HCl pH 8.0, 300 mM NaCl, 5% glycerol, and 200 mM imidazole). Subsequently, the 6His‐SUMO tag was cleaved using SUMO protease at a 6:100 μg (enzyme:tagged protein) mass ratio. The cleaved sample underwent a size‐exclusion chromatography using a Superdex 75 (16/60) column coupled to an AKTA FPLC system (GE). The column was pre‐equilibrated with SIRT1 buffer (20 mM HEPES pH 7.5, 150 mM NaCl, and 10% glycerol) and the sample was eluted isocratically with 1.2 CV. The fractions containing SIRT1 were collected, further analyzed by 15% SDS‐PAGE gel, and stored at −80°C.

### 2.2 PPARγ and SIRT1 Mutagenesis

We generated single‐point mutations in PPARγ (isoform 2) to (1) evaluate the impact of K268 and K293 acetylation on the binding strength of SIRT1:PPARγ by creating acetyl‐mimetic mutants (K268Q, K293Q), and (2) to validate the SIRT1 binding interface identified in MD simulation (L255R, L289D, V256R). The mutagenesis was performed using the QuickChange protocol. The primers used are listed in Table S1.

To assess the significance of each SIRT1 domain in PPARγ binding, we generated SIRT1 constructs lacking either NTD or CD. Deletion of SIRT1 NTD (ΔNTD) and CD (ΔCD) was performed on hSIRT1pET‐SMT3 1‐143CS vector. The PCR reaction was conducted in the presence of primers previously phosphorylated (Table S1) (10 U T4 PNK, 10 μM of each primer, PNK Buffer A, 10 mM ATP for a final reaction volume of 20 μL for 45 min at 37°C) using hSIRT1pET‐SMT3 1‐143CS vector as a template, under the same conditions as previously described [28]. Subsequently, the amplicons were circularized using T4 ligase (NEB) following the manufacturer's instructions. The samples were transformed into chemically competent bacteria and stored at −80°C. Confirmation of the deleted sequences of hSIRT1pET‐SMT3 1‐143CS vector was evidenced by Sanger sequencing.

### 2.3 Biophysical Characterization

Biophysical assays were performed to investigate possible alterations in the structure of PPARγ and SIRT1 constructs due to mutations. The hydrodynamic radius (*R*_H_) and polydispersity (%*P*_D_) were obtained through dynamic light scattering (DLS) experiments. For DLS, the experiments were performed in ZetasizerNano ZS instrument (Malvern). Data acquisition involved 9 μM of purified samples (PPARγ LBD WT, K268Q and K293Q) in their respective SEC buffer in a 1 mm quartz cuvette at 25°C with a 30‐s equilibrium. *R*_H_ and %*P*_D_ values were generated as an average of 100 reading accumulations by the Malvern software. Thermal stability of secondary structure was determined by circular dichroism (CD) and the stability of the tertiary structure via differential scanning fluorimetry (nano‐DSF). For CD, far‐ultraviolet (UV) CD spectra were obtained using a Jasco J‐810 spectropolarimeter (JASCO) coupled to a Peltier controller. Samples were diluted to 5 μM in a 0.1 mM phosphate buffer and were subjected to data collection. The far‐UV spectra were obtained from 190 to 260 nm at 4°C, with a scan speed of 50 nm/min with 20 accumulations using 1 mm quartz cuvettes (Hellma). Melting temperatures (*T*_m_) were determined by recording spectra at 222 nm from 20°C to 80°C (1°C/min) in triplicate and fitting the data to the Hill1 equation in the OriginPro 8.6 software. For the nano‐DSF, the measurements were carried out using a Tycho NT.6 instrument (NanoTemper Technologies). Data was collected in triplicate, and the intrinsic fluorescence ratio (350 nm/330 nm) was obtained over a temperature gradient ranging from 25°C to 90°C. Each capillary was loaded with 9 μM purified PPARγ LBD WT and mutants (K268Q, K293Q) in a 20 mM Tris pH 8, 150 mM NaCl, 5% glycerol, and 2 mM DTT buffer. The first derivative curves were normalized using OriginPro 8.6 software, and the inflection curves (*T*_i_) were calculated using the equipment software. Statistical analysis was performed using OriginPro 8.6 software, applying an unpaired t‐test to compare groups, with the significance level set as 95%.

We analyzed the sedimentation behavior of PPARγ:SIRT1 to obtain information about the stoichiometry of the complex using analytical ultracentrifugation through the method of sedimentation velocity (AUC‐SV). All samples were diluted in 20 mM HEPES, pH 7.5, 150 mM NaCl, 10% glycerol to a final concentration as follows: SIRT1 (10 μM), PPARγ (40 μM), and 10 μM of SIRT1 and 40 μM of PPARγ‐DL for the complex. The runs were conducted in the Beckman Coulter OptimaTM XL‐A ultracentrifuge at 20°C with a speed of 25.000 rpm. Sample sedimentation was monitored by measuring absorbance at 280 nm. Data processing utilized the Sedfit version 16.1c, employing the continuous distribution model *c*(*s*). The *s‐*values in this study are reported in Svedberg (S) units, which correspond to 10^−13^ s. The parameters for data processing were determined by SEDENTEP as follows: buffer density, 1.037; buffer viscosity, 0.0144; SIRT1 V_bar_, 0.742; PPARγ V_bar_, 0.743; SIRT1:PPARγ V_bar_, 0.741; and a f/f_o_, 1.37. The calculated parameters were determined using the Sednterp version 1.10 program, and the graphs were generated using GUISSI 1.2.1 [29, 30, 31].

To gain insight into the strength and specificity of PPARγ and SIRT1 interaction, we assessed their binding affinity by determining their dissociation constant (*K*_D_) through fluorescence polarization (FP) assay. We labeled SIRT1 with FITC (fluorescein isothiocyanate) at a molar ratio of 3 μM FITC to 1 μM SIRT1, SIRT‐ΔCD and SIRT1‐ΔNTD in alkaline buffer (50 mM HEPES, pH 8.0, 100 mM NaCl), to ensure the deprotection of primary amines, allowing for covalent attachment of FITC to lysine residues and the N‐terminus of SIRT1 [32]. The labeling process took place at 4°C for 3 h, and the excess probe was removed using a desalting column (HiTrap, 5 mL, GE). The serial dilution of PPARγ constructs and mutants was prepared in a buffer containing 50 mM HEPES, pH 8.0, 100 mM NaCl, 1 mM DTT, and the experiments were conducted in triplicates in 384‐well black plates (Greiner). The ligands were added with a 3‐fold molar excess of either SIRT1 or PPARγ concentration. Each point of the serial dilution (ranging from 12.5 μM to 0.02 μM) was incubated with 80 nM of the labeled‐SIRT1 constructs for 4 h at 4°C. Fluorescence anisotropy measurements were performed using a ClarioStar plate reader (BMG) with an emission wavelength of 520 nm and an excitation wavelength of 495 nm. The *K*_D_ was determined by fitting the fluorescence polarization values to the Hill1 equation using OriginPro 8.6 software [5, 28]. The statistical analysis was performed using the same software, an unpaired *t*‐test to compare groups with a significant level set as 95%.

### 2.4 In Silico Investigation

We built two models of the SIRT1:PPARγ complex, each distinguished by the positioning of the K268 or K293 residue of PPARγ, denoted as SIRT1‐_K268_PPARγ and SIRT1‐_K293_PPARγ, respectively. Utilizing x‐ray structures from the Protein Data Bank, we aligned the ligand binding domain of PPARγ (PDB ID: 1PRG, isoform 1) structure with the acetylated peptide in the SIRT1 crystal structure (PDB ID: 4ZZJ). Although the 1PRG structure refers to isoform 1—28 amino acids shorter at the N‐terminus than isoform 2—for consistency, this manuscript adopts the isoform 2 residue numbering [33]. The alignment of the sequences presented challenges due to the accessibility of PPARγ's K268 and K293 residues, and the necessary length of K268‐ and K293‐containing peptides of PPARγ for proper alignment. To address these challenges, we implemented a comprehensive six‐step protocol.

First, a structural alignment was conducted on three sirtuin structures with peptides docked in their active site (PDB IDs: 4ZZJ, 4IAO, and 2H4F, Figure S2A). The alignment revealed that residues −1, 0, +1, and +2 (with 0 denoting the acetylated residue) displayed the least invariant positions (Figure S2B). Concurrently, conformations of PPARγ's loops containing K268 or K293 were sampled using ClustENMD [34, 35]. The simulation utilized the first five global modes and ran through 10 generations. An energy minimization, followed by a heating‐up phase in implicit solvent, was executed to relax the conformers. The simulations were carried out on a GPU platform using OpenMM [36]. The maximum number of clusters was set at 20 during the initial generation and increased by 20 in each subsequent generation, reaching 200 clusters by the tenth generation. This resulted in a set of approximately 3000 configurations of PPARγ (Figure S2C).

Next, the sampled configurations of PPARγ were docked to SIRT1 by aligning positions −1, 0, +1, and +2 relative to K268 or K293 on the ac‐peptide of the SIRT1 structure (4ZZJ). This process produced two sets of around 3000 complex structures: one set with K268ac docked in the SIRT1 active site and the other with K293ac. It is important to mention that, for K268ac, we modified the SIRT1 structure by adjusting the SIRT1‐NTD(3HB) to enhance the docking space for PPARγ and minimize potential clashes.

Following the docking, a hierarchical clustering of the complexes was performed, reducing them from ~3000 to approximately 60 representative structures for each modeled complex. This was done using the cpptraj program from AmberTools23 [36, 37]. The representative structures with the lowest root mean square deviation (RMSD) relative to the peptide in the SIRT1 crystal structure were selected. From these structures, two from each modeled complex showed no significant clashes, and these were chosen to proceed with the model building.

To model the NTD(3HB)‐PPARγ interaction, we docked the NTD(3HB) onto the previously generated complexes (where NTD (3HB) had been removed) using ClusPro [35, 38, 39, 40]. Two distance restrictions (25 and 30 Å) were tested, yielding two models for the SIRT1‐_K268_PPARγ complex and four for the SIRT1‐_K293_PPARγ complex (Figure S2D). These models underwent 2‐microsecond MD simulation (for details see below). Out of the two representative models of the SIRT1‐_K268_PPARγ complexes, only one retained the NTD(3HB)‐PPARγ interaction along MD simulation. This model, which maintained the NTD(3HB)‐PPARγ interaction, was used for further analyses. Of four simulations for the SIRT1‐_K293_PPARγ complex, only one simulation was discarded due to NAD^+^ exploring unreactive configurations. The final chosen simulations for the SIRT1‐_K268_PPARγ and SIRT1‐_K293_PPARγ_1‐3_ complexes were used for contact analysis between the SIRT1‐NTD(3HB) and PPARγ. The model reaches stability at 1200 ns, as the RMSD analysis of the protein backbone using the first frame of production as reference (see Figure S3).

The Amber ff14SB force field was used to describe the protein residues and acetyl‐lysine [41]. TIP3P was the water model for solvation, creating a truncated‐octahedral box of 15 Å around the proteins [42]. The system was neutralized to a salt concentration of 0.15 M by adding Na^+^ and Cl^−^ ions [43, 44]. NAD^+^ parameters were taken from refs [45, 46], and the zinc AMBER Force Field (ZAFF) parameters were applied to Zn^2+^ and the cysteines in the first coordination shell [47, 48]. The MD simulations of the SIRT1:PPARγ complexes were equilibrated following the next protocol: an initial energy minimization of the solvent and protein side chains was performed using 2500 steps of steepest descent, followed by 2500 steps of conjugate gradient, applying positional restraints with a force constant of 100 kcal/mol Å^2^ to the protein backbone atoms (Cα, C, N, and O), as well as to the NAD^+^ molecules and the Zn^2+^ ion. This was followed by an unrestrained minimization of the entire system. Next, two simulations were run, a 2 ns NVT simulation followed by a 0.5 ns NPT simulation, both with the same restraints applied before. The final equilibration step was a subsequent 10 ns NPT simulation without any restraints. Finally, the production run was conducted over a 2 μs timeframe under constant pressure and temperature (NPT) conditions, with frames saved every 2 ns. The temperature control was maintained using the Stochastic version of the Berendsen thermostat [49] with a bath coupling of 1 ps, and pressure was controlled at 1 bar employing the Monte Carlo barostat algorithm [50] with a relaxation time of 1 ps. Hydrogen mass repartitioning [51] with the SHAKE algorithm [52, 53] allowed for a 4 fs timestep. All the MD simulations were performed using PMEMD.cuda [54].

To assess the equilibration of the MD simulations, we monitored three structural parameters throughout the trajectories. First, we evaluated the distances between the SIRT1 domains (NTD and CD) and PPARγ, as well as the inter‐domain angle defined by their geometric centers (Figure S4A). The inter‐domain angle was calculated using the geometric centers of the NTD, CD, and PPARγ domains, forming the NTD–CD–PPARγ angle, which reflects the relative orientation of the domains over time (Figure S4B). Additionally, we computed the Q (fraction of native contacts), as defined by Best, Hummer, and Eaton [55], to quantify the stability of interfacial contacts between PPARγ and each SIRT1 domain, using as reference the last frame of the simulation. The calculation was performed using MDanalysis and setting a cut‐off of 5 Å, a β of 5 Å, and a λ constant of 1.8 Å (Figure S4C) [56, 57]. These parameters showed that the structural equilibration for all systems was achieved at 1200 ns.

The contacts map and RMSD analysis were performed using the last 800 ns of simulation trajectories. MD analyses were conducted using CPPTRA [36], part of the AmberTools23 package [37].

## 3 Results

### 3.1 Mapping PPARγ and SIRT1 Binding Interfaces

The deacetylation of PPARγ's K268ac and K293ac by SIRT1 plays a crucial role in promoting insulin sensitivity. While SIRT1's deacetylase activity is central to these beneficial outcomes, the structural details of how SIRT1 binds to PPARγ during this process remain unclear [2]. In this study, we developed in silico experimentally supported binding models for SIRT1 binding to acetylated PPARγ at K268 (SIRT1‐_K268_PPARγ) or at K293 (K293 SIRT1‐_K293_PPARγ) to underscore their binding features. To guide the building process of these models, we mapped the key interfaces involved in PPARγ and SIRT1 binding by determining their dissociation constant (*K*_D_) using fluorescence polarization (FP) assay.

Rosiglitazone, a PPARγ activator, leads to the deacetylation of PPARγ at K268ac and K293ac. To assess whether Rosiglitazone influences PPARγ binding to SIRT1, we determined the *K*_D_ of PPARγ construct harboring DBD‐LDB (DL) and the SIRT1 construct harboring CD (catalytic core + ESA peptide) and NTD(3HB) in the presence of Rosiglitazone (Figure 1A) [19]. In the control condition (DMSO), PPARγ DL binds SIRT1 with a *K*_D_ of 1.90 ± 0.14 μM, which decreased to 0.69 ± 0.09 μM in the presence of Rosiglitazone (Figure 1B). This reduction in *K*_D_ indicates that Rosiglitazone enhances PPARγ's affinity for SIRT1, suggesting that the ligand‐dependent activation of PPARγ increases SIRT1 affinity. Next, we investigate whether the presence of the cofactor NAD^+^ modulates PPARγ binding. Contrary to Rosiglitazone, NAD^+^ presence resulted in a *K*_D_ of 1.67 ± 0.3 μM, comparable to the control (Figure 1B). These findings suggested that the structural changes caused by NAD^+^ did not significantly affect the overall binding of SIRT1:PPARγ in the context of FP assays. Given that Rosiglitazone binds to the ligand‐binding pocket in the LBD of PPARγ, we questioned whether the LBD is a key interface for SIRT1 binding. To answer this question, we deleted the DBD from the PPARγ DL construct and measured its *K*_D_ with SIRT1 (Figure 1A). The deletion increased the binding strength, with the shorter construct (harboring only LBD) displaying a *K*_D_ of 0.24 ± 0.03 μM, compared to the longer DL construct (Figure 1C). This result suggested that LBD serves as the major interface for SIRT1 binding, which is consistent with the observation that Rosiglitazone enhances SIRT1 binding affinity.

Following the identification of SIRT1 binding interfaces on PPARγ, we applied the same strategy to map PPARγ binding sites on SIRT1. Using FP assays, we assessed the *K*_D_ of truncated constructs of SIRT1: SIRT1‐ΔNTD (lacking NTD(3HB)) and SIRT1‐ΔCD (lacking catalytic core + ESA) with PPARγ‐LBD (Figure 1A). SIRT1 exhibited a *K*_D_ of 0.24 ± 0.03 μM, whereas the truncated forms presented a significant higher *K*_D_ value: 1.62 ± 0.28 μM for SIRT1‐ΔNTD and 6.55 ± 1.8 μM for SIRT1‐ΔCD (Figure 1D). Deletion of either domain impaired PPARγ binding highlighting the importance of CD and NTD(3HB) domains in maintaining SIRT1's binding strength. Notably, deletion of CD had a greater impact on binding compared to NTD(3HB), positioning the CD as the major contributor to the SIRT1:PPARγ interaction. Although the CD played a major role in binding, the NTD(3HB) contributed to the overall stability of the complex, as its deletion significantly weakened the interaction. Therefore, both SIRT1 CD and NTD(3HB) together constitute the PPARγ binding interface of SIRT1.

### 3.2 SIRT1 Forms a Heterocomplex With PPARγ

**FIGURE 2:**
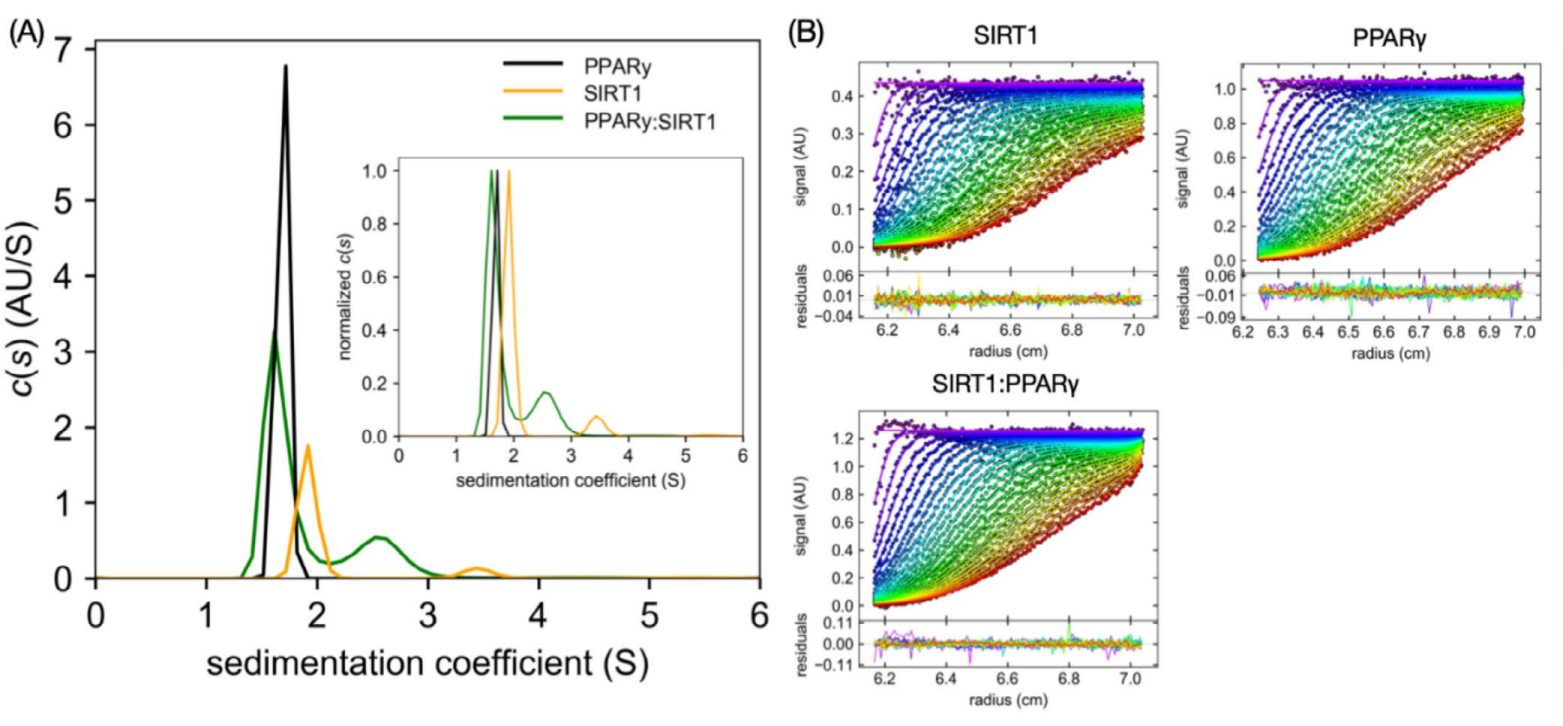
(A) *c*(*s*) distribution for PPARγ, SIRT1, and the SIRT1:PPARγ complex. A normalized *c*(*s*) distribution is inset in the main graph. (B) Raw sedimentation profiles of absorbance at 280 nm versus cell radius, along with the residual plot generated by the SEDFIT software.

After mapping the primary SIRT1:PPARγ binding interfaces, we sought to determine the stoichiometry of the proteins for modeling the complex. The stoichiometry was assessed using sedimentation velocity assay, through the method of analytical ultracentrifugation (AUC‐SV), which provides the sedimentation coefficient (*S*) and allows for the calculation of the protein size distribution in solution. To ensure all binding interfaces between SIRT1 and PPARγ were saturated, we used concentrations above the determined *K*_D_. The sedimentation coefficient distribution (*c*(*s*) vs. *S*) values of the samples are shown in Figure 2A. The raw data scans and the representative fits to a continuous *c*(*s*) distribution model are depicted in Figure 2B. PPARγ‐DL exhibited a singular *S* value of 1.69*S* ± 0.04, corresponding to a sedimentation particle mass of 35.6 ± 3.3 kDa, while SIRT1 showed two *S* values of 2.12*S* ± 0.12 and 3.41*S* ± 0.12, corresponding to sedimentation particle masses of 51.4 ± 5.7 kDa and 97.4 ± 11.3 kDa, respectively (Table 1). Thus, while the PPARγ‐DL sample consisted of monomeric forms, the SIRT1 sample contained both monomeric and dimeric forms. In the SIRT1:PPARγ‐DL sample, two peaks were observed: one at 1.74*S* ± 0.12 and another at 2.89*S* ± 0.21, corresponding to sedimentation particle masses of 38.06 ± 4.6 kDa and 73.6 ± 6.6 kDa, respectively (Table 1). The 1.74*S* peak corresponded to the unbound state, likely driven by high PPARγ concentration. The 2.89*S* ± 0.21 peak matched the combined masses of the monomeric SIRT1 (51.4 ± 5.7 kDa) and PPARγ‐DL (35.6 ± 3.3 kDa) in a 1:1 ratio (~77 kDa), indicating the formation of a heterodimeric complex. Additionally, the absence of the SIRT1 dimer peak in the complex sample suggested that PPARγ promotes the dissociation of the SIRT1 dimer, which may indicate a structural mechanism of SIRT1:PPARγ assembly.

**TABLE 1:** Complex characterization by AUC‐SV experiment.

	Peak	*S*_w_ ^a^	*S*_w20_ ^b^	*R*_H_ (nm)	%^c^	*M*_w_ (kDa)
PPARγ‐DL (42.4 kDa)	1	1.69*S* ± 0.04	2.72 ± 0.04	3.03	96	35.6 ± 3.3
SIRT1 (45 kDa)	1	2.12*S* ± 0.12	3.41 ± 0.12	3.41	81.1	51.4 ± 5.7
	2	3.24*S* ± 0.27	5.28 ± 0.27	4.22	19.4	97.4 ± 11.3
SIRT1:PPARγ‐DL (87.4 kDa)	1	1.74*S* ± 0.12	2.89 ± 0.2	3.12	77.1	38.06 ± 4.6
	2	2.89*S* ± 0.21	4.39 ± 0.21	4.00	21.9	73.6 ± 6.6

*Note*: Mean ± standard deviation (SD).

^a^ Svedberg unit.

^b^ Svedberg unit (20°C).

^c^ Corresponding percentage of signal in the sample.

### 3.3 The PPARγ K268 and K293 Acetyl‐Mimetic Modulates SIRT1 Binding

The insulin‐sensitizing outcomes due to PPARγ activation require the deacetylation of K268ac and K293ac, which are in the loop regions of PPARγ's‐LBD: α1‐β1 and α2′‐α3, respectively. These loop regions exhibit greater dynamics and flexibility, making them susceptible to conformational changes in response to chemical modification, such as acetylation. Consequently, K268ac and K293ac could alter loop dynamics, potentially affecting the protein's structural stability and binding affinity to protein partners. This led us to investigate whether PPARγ K268 and K293 acetylation would influence SIRT1 binding. To simulate the acetylated state of the protein without requiring enzymatic activity, we generated two PPARγ acetyl‐mimetic mutants by mutating the positivelycharged lysine at K268 and K293, creating the K268Q and K293Q mutants [58].

**FIGURE 3:**
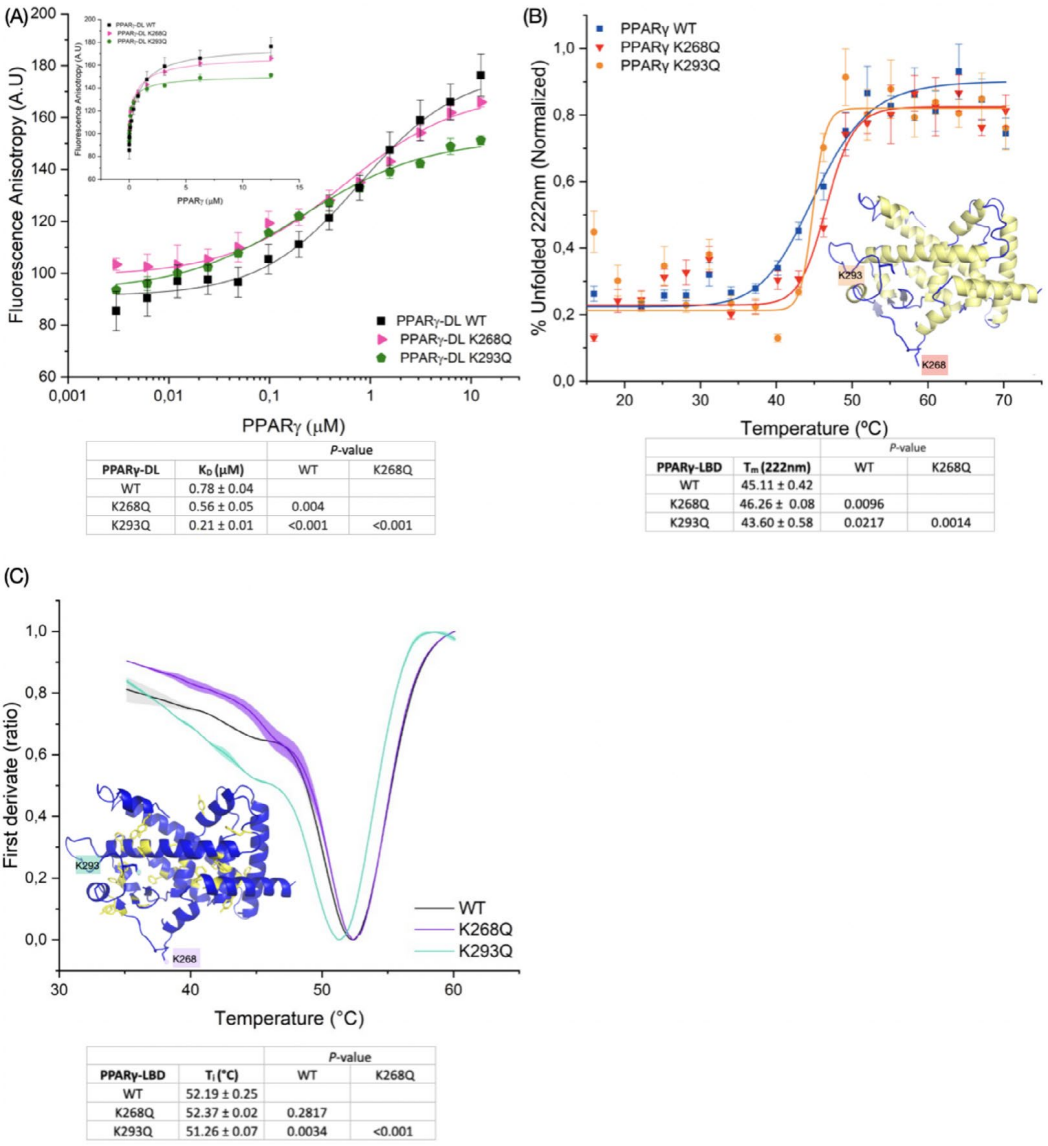
(A) FP curves of PPARγ‐DL WT and acetyl‐mimetic K268Q and K293Q with labeled‐SIRT1. Linear scale graphs and *K*_D_ values table are inserted within the main FP graph. (B) CD thermal denaturing curves following the signal at 222 nm. *T*_m_ were determined for PPARγ‐LBD WT and acetyl‐mimetics mutants. (C) *T*_i_ determined by fluorescence decay ratio 350 nm/330 nm of PPARγ‐LBD WT and acetyl‐mimetic mutants. A table with *T*_i_ and *T*_m_ values are below each graph, *p* values from the unpaired *t*‐test are indicated in the same table. The schematic representation of PPARγ is shown in blue, with monitored structures during thermal denaturation highlighted in yellow as inserts: Secondary structure (B) and tryptophan and tyrosine residues (C).

The mutations did not impact on the hydrodynamic radius (*R*_H_), polydispersity percentage (P_D_%) or CD spectra profile of the samples, confirming that the introducing of these mutations did not alter the secondary or tertiary structure of constructs (Figure S5). Figure 3A shows FP curves for PPARγ acetyl‐mimetics mutants against labeled‐SIRT1. Both K268Q and K293Q mutants exhibited *K*_D_ values within the nanomolar range, similar to PPARγ WT. The maintenance of *K*_D_ suggested that PPARγ acetylation did not disrupt SIRT1 binding; rather, it maintained the affinity between the two proteins. Notable, K293Q had a *K*_D_ of 0.21 ± 0.01 μM while K268Q had a *K*_D_ of 0.56 ± 0.05 μM. This indicated that the acetyl‐mutation enhanced SIRT1 binding, aligning with the major role of LBD region mediating this interaction. However, the K293ac played a more significant role in SIRT1 binding than K268, as indicated by lower *K*_D_ of K293Q mutant. Thus, using acetyl‐mimetic mutants, we observed that PPARγ acetylation at K293 and K268 enhances SIRT1 binding, with binding affinity varying depending on the acetylation site. Our findings align with expectations, as SIRT1 naturally interacts with acetylated substrates, and the acetyl‐mimetic mutants employed in this study preserve this characteristic.

The variation in binding strength observed in response to acetyl‐mimetic mutation prompted us to explore whether these differences in *K*_D_ correlate with changes in PPARγ's structure caused by the chemical alteration of lysine residues. We assessed the impact of K268 and K293 acetylation on PPARγ's structure by analyzing the thermal stability of PPARγ acetyl‐mimetics K268Q and K293Q, monitoring changes in tertiary and secondary structures under thermal denaturation conditions. Figure 3B presents the melting curves from the CD experiment following the signal decay at 222 nm. The acetyl‐mimetics showed a distinct *T*_m_ values compared to WT. The K268Q had a *T*_m_ of 46.26°C ± 0.08°C, the K293Q had a *T*_m_ of 43.60°C ± 0.58°C, while WT had a *T*_m_ of 45.11°C ± 0.42°C. Interestingly, the effect of mutation was different: K268Q increases *T*_m_, while K293Q decreases it, indicating a *T*_m_ dependency on the positioning in response to the acetyl‐mimetic mutations. Additionally, intrinsic fluorescence decay, named inflection temperature (*T*_i_), revealed the same trend. The *T*_i_ of WT and K268Q were 52.19°C ± 0.25°C and 52.37°C ± 0.02°C, respectively, while K293Q had a *T*_i_ of 51.26°C ± 0.07°C (Figure 3C). These data collectively suggested that acetyl‐mimetic mutations modulated PPARγ thermal stability, with K268Q primarily stabilizing the secondary structure and K293Q destabilizing both secondary and tertiary structures of PPARγ.

Therefore, our results show PPARγ acetylation would affect SIRT1 binding affinity with distinct effects at different lysine residues. While K268 acetylation increased PPARγ's thermal stability, K293 acetylation decreased it. The K293 mutation had a more pronounced impact on both SIRT1:PPARγ binding affinity and PPARγ's thermal stability compared to the K268 mutation. This indicates that the acetyl‐mimetic mutation at positions 268 and 293 affects PPARγ's binding interaction and thermal stability in distinct ways, highlighting the differential roles of K268 and K293 residues in PPARγ stability and its interaction with SIRT1.

### 3.4 PPARγ Binds to SIRT1 NTD(3HB) and CD Regions

This work aimed to elucidate binding interfaces of PPARγ:SIRT1 by generating experimentally based models of the complex. Our findings indicated that SIRT1 forms a heterodimer with PPARγ, with the PPARγ‐LBD and the SIRT1 NTD(3HB) and CD regions serving as primary binding domains. We also suggested that acetylated PPARγ, specifically K268ac and K293ac, modulated SIRT1 binding in distinct ways. These features were input into in silico studies to generate the models: SIRT1‐_K268_PPARγ and SIRT1‐_K293_PPARγ. The assembling of the complex followed a hierarchical approach, considering the *K*_D_ of PPARγ‐LBD and the SIRT1 constructs. The first step involved docking the PPARγ‐LBD to the SIRT1 CD region. This process was guided by the ac‐peptide length and positioning of the acetylated residue. However, the absence of SIRT1 structures in complex with native substrates led an initial investigation to determine the length and position of PPARγ acetyl‐lysine peptides to be considered for docking. To address this, we aligned structures of SIRT1's catalytic core in complex with acetyl‐peptides (Figure S2A). The peptides positions −1, 0 (Ac‐residue), +1, and +2 exhibited minimal variability and were, therefore, considered for docking the K268ac and K293ac peptides to the catalytic core of SIRT1 (Figure S2B). Additionally, we suggested that acetylation modulated the thermal stability of PPARγ, likely due to changes in conformation of K268‐ and K293‐containing loops. To account the structural variations of the peptides to the models, we performed a conformational sampling of the α1‐β1 and α2′‐α3 loops containing the K268ac and K293ac, respectively (Figure S2C). Once the SIRT1 CD was accurately positioned, we incorporated the SIRT1‐NTD(3HB) into the model. Given that the 3HB region of NTD is the only available crystal structure, we only considered 3HB for docking. NTD(3HB) is connected to CD through a flexible link region, in which we applied this distance as a restraint for NTD(3HB) docking [19] (Figure S2D). Two docking calculations were performed, applying distances restraint between the C‐terminal of the NTD(3HB) and the N‐terminal of the CD of SIRT1 (see Section 2 for more details about model construction).

**FIGURE 4:**
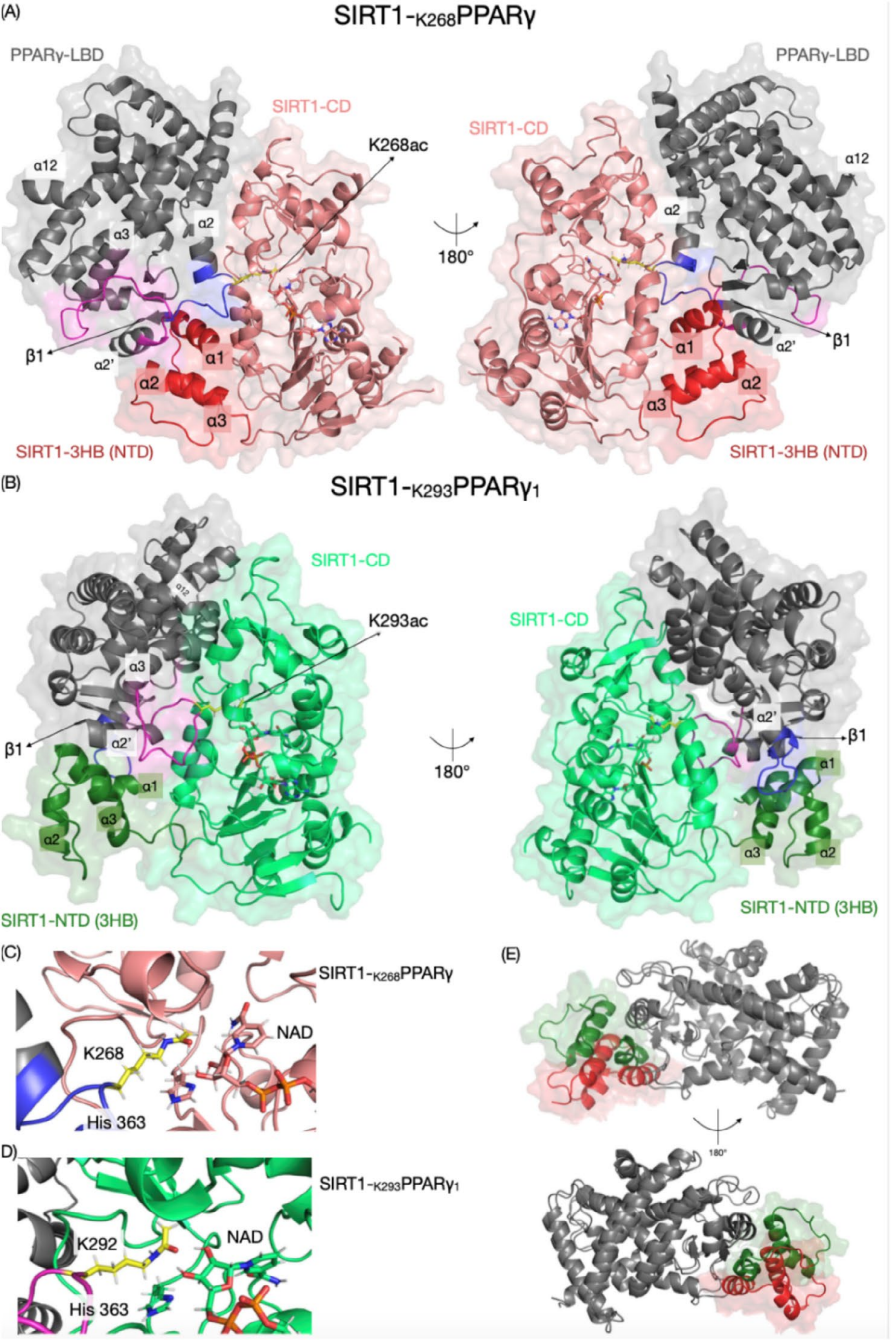
(A) Model of SIRT1‐_K268_PPARγ (red) and (B) model of SIRT1‐K_293_PPARγ_1_ (green). The models SIRT1‐K_293_PPARγ_2‐3_ are shown in Figure S6. In both models, the PPARγ LBD (gray) binds to a region of SIRT1 formed by CD and NTD(3HB). (C) Positioning of K268ac and (D) K293ac in the SIRT1 catalytic core, with both residues located near to catalytic histidine and NAD^+^. (E) Superimposition of PPARγ binding interface of SIRT1's NTD(3HB) from SIRT1‐_K268_PPARγ and SIRT1‐_K293_PPARγ_1_ models. The acetylated lysine's are colored yellow, while α1‐β1 and α2′‐α3 loops are shown in blue and pink, respectively.

At the end, we generate one model for K268, SIRT1‐_K268_PPARγ, and three models for K293, SIRT1‐_K293_PPARγ_1‐3_. Here, we present SIRT1‐_K293_PPARγ_1_ as a representative model for K293, while the other two are shown in Figure S6. The SIRT1‐_K268_PPARγ and SIRT1‐_K293_PPARγ_1_ models showed the LBD of PPARγ clamped by NTD(3HB) and CD regions of SIRT1 (Figure 4A,B). These models also revealed that the binding interface of PPARγ on SIRT1 was formed by CD and NTD(3HB) domains. Furthermore, the models indicated that K268ac and K293ac residues of PPARγ were positioned near the nicotinamide moiety of NAD^+^ and the active histidine within the catalytic core of SIRT1 (Figure 4C,D). A comparative analysis of models showed that NTD(3HB) region bound to the same PPARγ region in both models, suggesting a common SIRT1 binding interface on PPARγ (Figure 4E). However, the model also revealed differences in binding interfaces. In the SIRT1‐_K268_PPAR model, SIRT1 CD made exclusive contacts with α1‐β1 loop, a feature absent in the SIRT1‐_K293_PPARγ models. In contrast, in the SIRT1‐_K293_PPARγ models, the α2′‐α3 loop bound to NTD(3HB) of SIRT1, suggesting that α2′‐α3 loop may serve as an additional binding interface for SIRT1 docking. Thus, the binding interface of SIRT1 on PPARγ was structured by NTD(3HB) and CD regions of SIRT1, with the NTD(3HB) as a common binding site for both PPARγ acetylated states.

### 3.5 Mapping Putative Residue of NTD(3HB) Binding Interface of PPARγ

The presented models offered an initial snapshot of PPARγ:SIRT1 binding interactions, highlighting their binding interfaces. While the SIRT1 CD was positioned differently in relation to the PPARγ K268ac and K293ac peptide, the NTD(3HB) consistently assumed the same position in both the SIRT1‐_K268_PPARγ and SIRT1‐_K293_PPARγ_1‐3_ models. This indicates that SIRT‐NTD(3HB) serves as a common binding interface for PPARγ, emphasizing its role in substrate anchoring. To further explore the NTD(3HB) binding interface, we sought to identify the putative residues in SIRT1's NTD(3HB) regions that are crucial for PPARγ binding.

**FIGURE 5:**
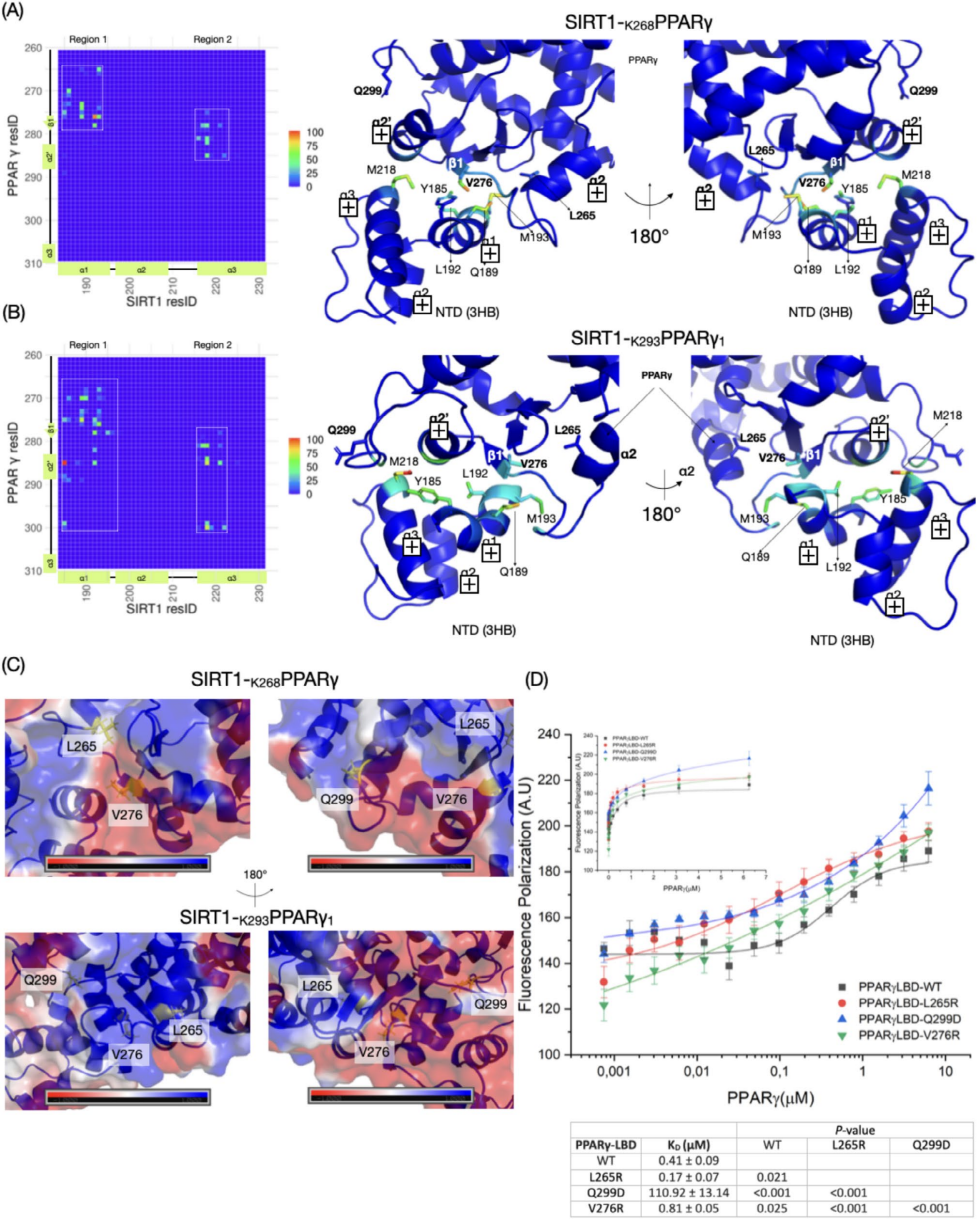
Visualization of the interaction between SIRT1‐NTD(3HB) and PPARγ for SIRT1‐_K268_PPARγ (A) and SIRT1‐_K293_PPARγ_1_ (B) models. The SIRT1‐_K293_PPARγ_2‐3_ contact maps are shown in Figure S7. The crystal structures are colored according to their respective contact maps on the left. The frequency of contact maps shows the persistence of interaction along the MD between SIRT1‐NTD(3HB) and PPARγ residues. The distance cut‐off was set to 5 Å, considering the minimum distance between pairs of residues. The numbers on the *y*‐axis and *x*‐axis indicate the amino acid positions within the PPARγ (LBD) and SIRT1‐NTD(3HB) structures, respectively. The color intensity of each cell represents the frequency of contact between the corresponding PPARγ (LBD) and SIRT1‐NTD(3HB) residues (C) PPARγ electrostatic potential of the PPARγ binding interface for SIRT1‐NTD(3HB), calculated by APBS electrostatic software [59]. (D) FP assay with labeled‐SIRT1 and PPARγ‐LBD mutants targeting the SIRT1‐NTD(3HB) binding interface. *K*_D_ values included as inset.

To do this, we performed MD simulations between SIRT1 NTD(3HB) and PPARγ LBD in SIRT1‐_K268_PPARγ and SIRT1‐_K293_PPARγ_1‐3_ models. Across the simulations, the SIRT1‐_K268_PPARγ model and the three simulations of the SIRT1‐K_293_PPARγ models exhibited stability, suggesting that the acetylated forms of PPARγ maintain their structural integrity throughout the simulations. The contact maps for the SIRT1‐_K268_PPARγ and SIRT1‐K_293_PPARγ_1_ are shown in Figure 5A,B, respectively. The simulation for SIRT1‐K_293_PPARγ_2‐3_ is shown in Figure S7. These maps revealed two clusters of residues pairs coordinated by α1 (region 1) and α3 (region 2) within NTD(3HB). While these regions shared residue pairs in both models, the SIRT1‐_K293_PPARγ maps had additional numbers of residues pair in α2′‐α3 loop region of PPARγ. Among the identified residue pairs, we highlighted V276 in β1 of PPARγ as a putative key residue for SIRT1 docking. V276 consistently interacts with Q189, L192, and M193 of regions 1 and 2 in both models. Additionally, we observed residue pairs that were specific to each model. L265 in the SIRT1‐K_268_PPARγ model exclusively contacted M193 (α1) of SIRT1, while Q299 in the SIRT1‐_K293_PPARγ_1_ model uniquely interacted with Y185 and M218 (α3) of SIRT1.

To validate the importance of these PPARγ residues, we generated PPARγ mutants and determined their *K*_D_ using FP assay. Electrostatic potential mapping of the PPARγ structure revealed that L265 and Q299 are located in regions of predominantly positive electrostatic potential, whereas V276 lies within a negatively charged region (Figure 5C). Based on this analysis, mutations were introduced to alter the electrostatic environment of these residues: The L265R and V276R for positive charge, and Q299D for negative charge. These mutations did not significantly affect *R*_H_ or %*P*_D_ of PPARγ, indicating no major changes to the protein's tertiary structure making them suitable for FP binding assay (Figure S5). In the FP assay, the L265R and V276R exhibited *K*_D_ values in nanomolar range (0.17 ± 0.07 μM and 0.81 ± 0.05 μM, respectively), while the Q299D mutant displayed a *K*_D_ in a micromolar range of 110.92 ± 13.14 μM. Comparative analysis with PPARγ WT revealed that these mutations modulate SIRT1 binding differently. The L265R mutation enhanced SIRT1 binding, whereas V276 and Q299 weakened it, with Q299D mutation critical for SIRT1 binding. Although the effects varied among mutants, these results confirm that PPARγ residues L265 and V276 contribute to binding, while Q299 is essential for the SIRT1‐NTD(3HB) interaction, consistent with the proposed models.

## 4 Discussion

SIRT1 mediates insulin sensitization by deacetylating PPARγ at positions K268 and K293 [2]. Through the integration of biophysical methods and in silico studies, we generated an experimental‐based model for SIRT1 anchoring to acetylated PPARγ at these positions. The SIRT1‐_K268_PPARγ and SIRT1‐_K293_PPARγ_1‐3_ models revealed that SIRT1 anchors PPARγ via an interface formed by SIRT1's NTD(3HB) and CD regions, which collectively regulates the binding strength of acetylated PPARγ. This arrangement ensures a stable and specific interaction between SIRT1 and PPARγ, highlighting the distinct roles of these domains in substrate recognition. Notable, the transcriptional factor RXR and the co‐repressor protein NCoR, bind to PPARγ at a separate interface, which are critical for transcriptional regulation and repression, respectively [60] (Figure S8). This suggests that SIRT1 can anchor to PPARγ independently of its interaction with RXR or NCoR, potentially allowing simultaneous regulation of PPARγ activity by SIRT1 without interfering with its transcriptional or repressive functions. Moreover, although crystal structures of the full PPARγ:RXR:DNA complex exist (e.g., PDB ID: 3DZY), small angle x‐ray scattering (SAXS) analyses have shown that the complex adopts a more extended conformation in solution, with flexible linkers separating the DBDs and LBDs [60, 61, 62]. These observations support our use of an isolated LBD for modeling, as including the full complex would not only increase computational demand but also introduce uncertainty due to the high conformational flexibility of the full assembly.

The models also demonstrated that both SIRT1‐_K268_PPARγ and SIRT1‐_K293_PPARγ_1‐3_ share a common binding interface, involving the 3HB region in NTD, and PPARγ's residues Q299, V276, and L265 at α2‐α3 region of the PPARγ‐LBD. While the SIRT1‐_K268_PPARγ and SIRT1‐_K293_PPARγ models show commonalities, they also exhibited notable differences. Especially, the α1‐β1‐α2 region of the PPARγ that adopts different surface orientations in each model, exposing distinct residues of PPARγ to the SIRT1‐NTD(3HB). These observations align with previous findings that deletion of the NTD(3HB) region in SIRT1 impairs its activity in vivo [63]. Thus, based on our models, the NTD(3HB) region in SIRT1 may enhance substrate anchoring by providing an extra binding interface, potentially augmenting SIRT1's activity towards the substrate.

PPARγ acetyl‐mimetics mutants improved SIRT1 binding, but the extent of this improvement differed. The acetyl‐mimetics constructs of PPARγ (K268Q and K293Q) contributed to SIRT1 binding, but they modulate it in different ways [2]. This variation was attributed to changes in PPARγ thermostability resulting from the neutralization of lysine charge by acetylation [64]. Specifically, the K293Q mutant, unlike K268Q, displayed a lower *T*_m_ compared to the WT. This suggests that K268ac and k293ac might differently affect SIRT1 binding strength by altering PPARγ intramolecular interaction and thermostability.

SIRT1 is known to form oligomeric structures in solution, though the role of these structures in its activity remains unclear. The current model supports the idea that the monomeric state of SIRT1 exhibits higher activity than its oligomeric state [21]. We demonstrated that SIRT1 formed a homodimer in solution and PPARγ was predominantly monomeric, as detected by AUC‐SV analysis [61]. Interestingly, the presence of PPARγ led to dissociation of the SIRT1 homodimer, suggesting that substrate binding promotes the transition from a heterocomplex with PPARγ [27, 65]. Notably, deletion of NTD(3HB) in SIRT1 resulted in a decrease in its *R*_H_, with ΔCD construct showing a *R*_H_ like SIRT1. This implies that the NTD may play a role in dimerization, while ΔCD construct may adopt an alternative conformation in solution, accounting for the observed *R*_H_ [2]. According to our models, the dissociation of the SIRT1 homodimer and the subsequent formation of a heterocomplex with PPARγ could be mediated by SIRT1‐NTD(3HB), underscoring the importance of this region for substrate anchoring and SIRT1 oligomerization.

This work contributed to the understanding of PPARγ and SIRT1 binding interfaces by generating experimental‐based models of acetylated PPARγ at K268 and K293 with SIRT1. We highlighted the pivotal role of the SIRT1 NTD(3HB) and CD interface in PPARγ anchoring, identifying the SIRT1‐NTD(3HB) as a shared binding interface in both the SIRT1‐_K268_PPARγ and SIRT1‐_K293_PPARγ models. These findings provided new insights into SIRT1 interaction with PPARγ, revealing a novel binding interface that could serve as a promising target for developing drugs aimed at improving insulin sensitivity.

## Author Contributions

**Caique Camargo Malospirito:** investigation, writing – original draft, methodology, formal analysis, data curation, validation, conceptualization. **Gabriel Ernesto Jara:** data curation, formal analysis, methodology, writing – review and editing, investigation, software. **Víctor Ulian Antunes:** investigation, formal analysis, methodology, data curation. **Giovanna Blazutti Elias:** investigation, methodology, formal analysis, data curation. **Marieli Mariano Goncalves Dias:** investigation, methodology, formal analysis, writing – review and editing. **Fernanda Aparecida Heleno Batista:** investigation, visualization, methodology. **Paulo Sergio Lopes de Oliveira:** conceptualization, writing – review and editing, formal analysis, data curation, supervision, software. **Ana Carolina Migliorini Figueira:** conceptualization, funding acquisition, validation, project administration, supervision, resources, writing – review and editing.

## Conflicts of Interest

The authors declare no conflicts of interest.

## Supporting information


**Table S1.** Primers used for the mutation of PPARγ and SIRT1 constructs.
**Figure S1.** In vitro acetylation/deacetylation assay.
**Figure S2.** K268 and K293 peptide study for SIRT1 docking.
**Figure S3.** RMSD along the MD simulation time for the models SIRT‐_K268_ PPARy, SIRT1‐_K293_PPARy_1_, SIRT1‐_K293_PPARy_2_, and SIRT11‐_K293_PPARy_3_.
**Figure S4.** Time evolution of structural parameters describing the SIRT1–PPARγ complex.
**Figure S5.** Biophysical Characterization of protein constructs.
**Figure S6.** Model of SIRT1‐K_293_PPARγ_2_ and model of SIRT1‐K_293_PPARγ_3_.
**Figure S7.** Contact map from MD simulation of SIRT1‐K268PPARγ and SIRT1‐K293PPARγ model.
**Figure S8.** SIRT1 is bound to a distinct interface of the RXR and NCoR.

## Data Availability

The data that support the findings of this study are available on request from the corresponding author. The data are not publicly available due to privacy or ethical restrictions.
