# Supplementary material for: The SIRT1 N‐Terminal Domain as a Common Binding Interface for PPARγ Anchoring
Source: Proteins. 2025 Jul 17;93(12):2167–81. doi: 10.1002/prot.70022 (PMC12594183; doi:10.1002/prot.70022)
Supplement: Supplementary file 1 — Table S1. Primers used for the mutation of PPARγ and SIRT1 constructs. Figure S1. In vitro acetylation/deacetylation assay. Figure S2. K268 and K293 peptide study for SIRT1 docking. Figure S3. RMSD along the MD simulation time for the models SIRT‐K268 PPARy, SIRT1‐K293PPARy1, SIRT1‐K293PPARy2, and SIRT11‐K293PPARy3. Figure S4. Time evolution of structural parameters describing the SIRT1–PPARγ complex. Figure S5. Biophysical Characterization of protein constructs. Figure S6. Model of SIRT1‐K293PPARγ2 and model of SIRT1‐K293PPARγ3. Figure S7. Contact map from MD simulation of SIRT1‐K268PPARγ and SIRT1‐K293PPARγ model. Figure S8. SIRT1 is bound to a distinct interface of the RXR and NCoR. [file PROT-93-2167-s001.docx]

The SIRT1 N-terminal domain as common binding interface for PPARγ anchoring

*Caique Camargo Malospirito^12 †^, Gabriel Ernesto Jara^1 †^, Víctor Ulian Antunes^1^, Giovanna Blazutti Elias^13^, Marieli Mariano Goncalves Dias ^2^, Fernanda Aparecida Heleno Batista^4,^ Paulo Sergio Lopes de Oliveira^1^, Ana Carolina Migliorini Figueira^12*^*

^1^Brazilian Biosciences National Laboratory (LNBio), Brazilian Center for Research in Energy and Materials (CNPEM), Campinas, Brazil.

^2^ Graduate Program in Molecular and Morpho functional Biology, Institute of Biology, State University of Campinas (Unicamp), Campinas, Brazil.

3 Graduate Program in Pharmaceutical Sciences, Faculty of Pharmaceutic Sciences, State University of Campinas (Unicamp), Campinas, Brazil.

4 Molecular Investigation Laboratory in Cardiology, Institute Dante Pazzanese of Cardiology, São Paulo, Brazil.

KEYWORDS

SIRT1, PPARγ, insulin resistance, protein biochemistry, molecular modeling, protein biophysics.

Supplementary Figure S1
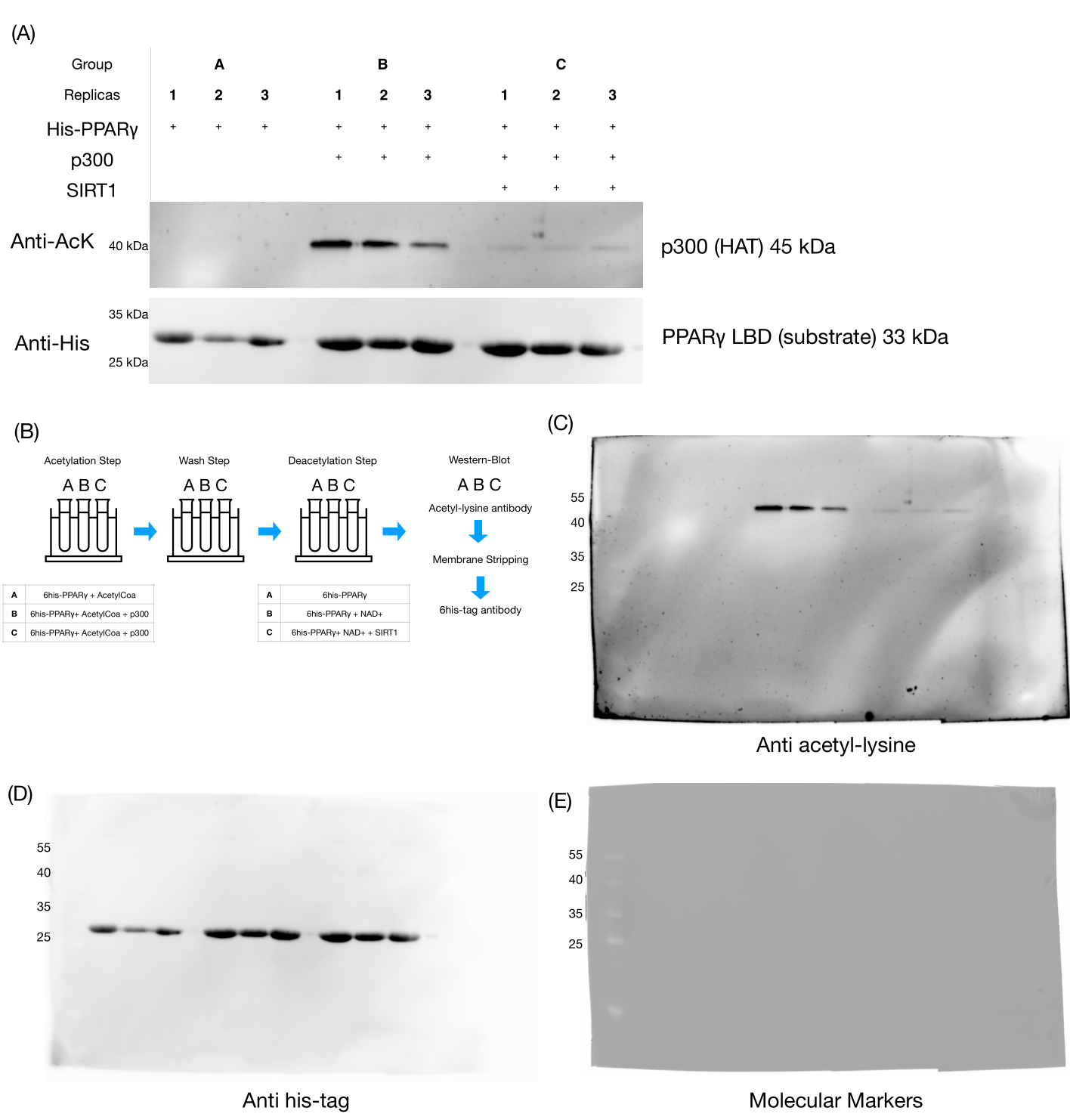


Supplementary Figure S1. SIRT1 deacetylation activity using native PPARγ LBD construct as the acetyl-substrate. (A) Western-Blot analysis of acetylation/deacetylation assays. Group A represents the negative control; group B represents the maximum acetylation levels and C group represents the acetylation levels after SIRT1 treatment. Uncropped membranes of the western-blot analysis are shown using (A) anti-acetyl lysine antibody (B) Anti-his antibody, and (D) the molecular weight markers photographed under visible light. The experiment war performed as follows:

***In-vitro acetylation/deacetylation assay -*** The *in-vitro* PPARγ deacetylation assay involved an initial acetylation step followed by SIRT1-mediated deacetylation, with deacetylation rates determined by sample C. Samples A and B served as control negative for negative acetylation and full PPARγ acetylation, respectively, ensuring that all samples underwent the same steps.

For PPARγ acetylation, 20 μM of 6xHis-tagged PPARγ was mixed with 0.5 μg of p300 (Enzo Bioscience - BML-SE451), 1 mM Acetyl-CoA, and 10 μL of cobalt-based resin in acetylation buffer (50 mM Tris pH 7.5, 150 mM NaCl, 1 mM PMSF, 1 mM DTT, and 10% glycerol) and incubated for 2 hours at 30°C. The sample was then transferred to filtered spin columns, centrifuged for 2 minutes at 500 rpm, and washed twice with acetylation buffer containing 5 mM imidazole. The resin-bound 6xHis-PPARγ was transferred to a new microtube and incubated with deacetylation components: 1 mM NAD^+^, 400 nM SIRT1 in PBS (pH 7.4), 1 mM DTT, and 2% glycerol for 1 hour at 37°C. Imidazole was added to a final concentration of 300 mM, and the flow-through was quenched with SDS-PAGE sample buffer.

Acetylation levels were analyzed by Western blot. Samples were run on a 15% SDS-PAGE gel, transferred to a 0.44 μm nitrocellulose membrane (300 mA, 1 hour, 4°C), blocked with 3% BSA in TBS-T, and washed with TBS-T 3-times. The membrane was incubated overnight with an anti-acetyl lysine antibody (Ac-K2-100 – 1:500), followed by a secondary antibody according to the manufacturer's instructions. The anti-acetyl lysine antibody was removed using a mild strip solution (25 mM glycine-HCl pH 2, 1% SDS), and the membrane was incubated with an anti-His tag antibody (A7058-1VL – 1:50000) according to the manufacturer's instructions. Visualization was performed using Clarity Western ECL substrate (Bio-Rad) and the ChemiDoc system (Bio-Rad). The result from the assay is shown in Supplementary Figure S1. The PPARγ-LBD did not display a band when probed with anti-acetyl antibody (S1C), whereas the bands visible in S1C corresponded to the p300 HAT domain. These bands showed a reduction in acetylation levels after SIRT1 treatment, demonstrating that the SIRT1 construct used in this study possesses enzymatic activity.

**Supplementary Table 1. Primers** used for the mutation of PPARγ and SIRT1 constructs.

| Sample | | DNA Sequence |
| --- | --- | --- |
| PPARγ K268Q | Forward | GATTTGTCTGTTGTCTGTCCTGTCAAGATCGCCCT |
|  | Reverse | AGGGCGATCTTGACAGGACAGACAACAGACAAATC |
| PPARγ K293Q | Forward | CTGCAGGGGGGTGATGTGCTGGAACTTGATTTTATCTTCTCC |
|  | Reverse | GGAGAAGATAAAATCAAGTTCCAGCACATCACCCCCCTGCAG |
| PPARγLBD L237R | Forward | GCAAAGGCGAGGGCGATCAGGACAGGAAAGACAACAGAC |
|  | Reverse | GTCTGTTGTCTTTCCTGTCCTGATCGCCCTCGCCTTTGC |
| PPARγLBD V248R | Forward | ACAGACAAATCACCATTCCGTATCTATGACATGAAT |
|  | Reverse | ATTCATGTCATAGATACGGAATGGTGATTTGTCTGT |
| PPARγLBD Q271D | Forward | AAACACATCACCCCCCTGGACGAGCAGAGCAAAGAGGTG |
|  | Reverse | CACCTCTTTGCTCTGCTCGTCCAGGGGGGTGATGTGTTT |
| SIRT1 ΔCD | Forward | TGAGTCGACAAGCTTGCGGCC |
|  | Reverse | TTTTGGTGGTTCTGAAAGGAT |
| SIRT1 ΔNTD | Forward | ATTAATACAATTGAAGATGCTGTG |
|  | Reverse | TCGGGATCCACCAATCTGTTC |

Supplementary Figure S2.


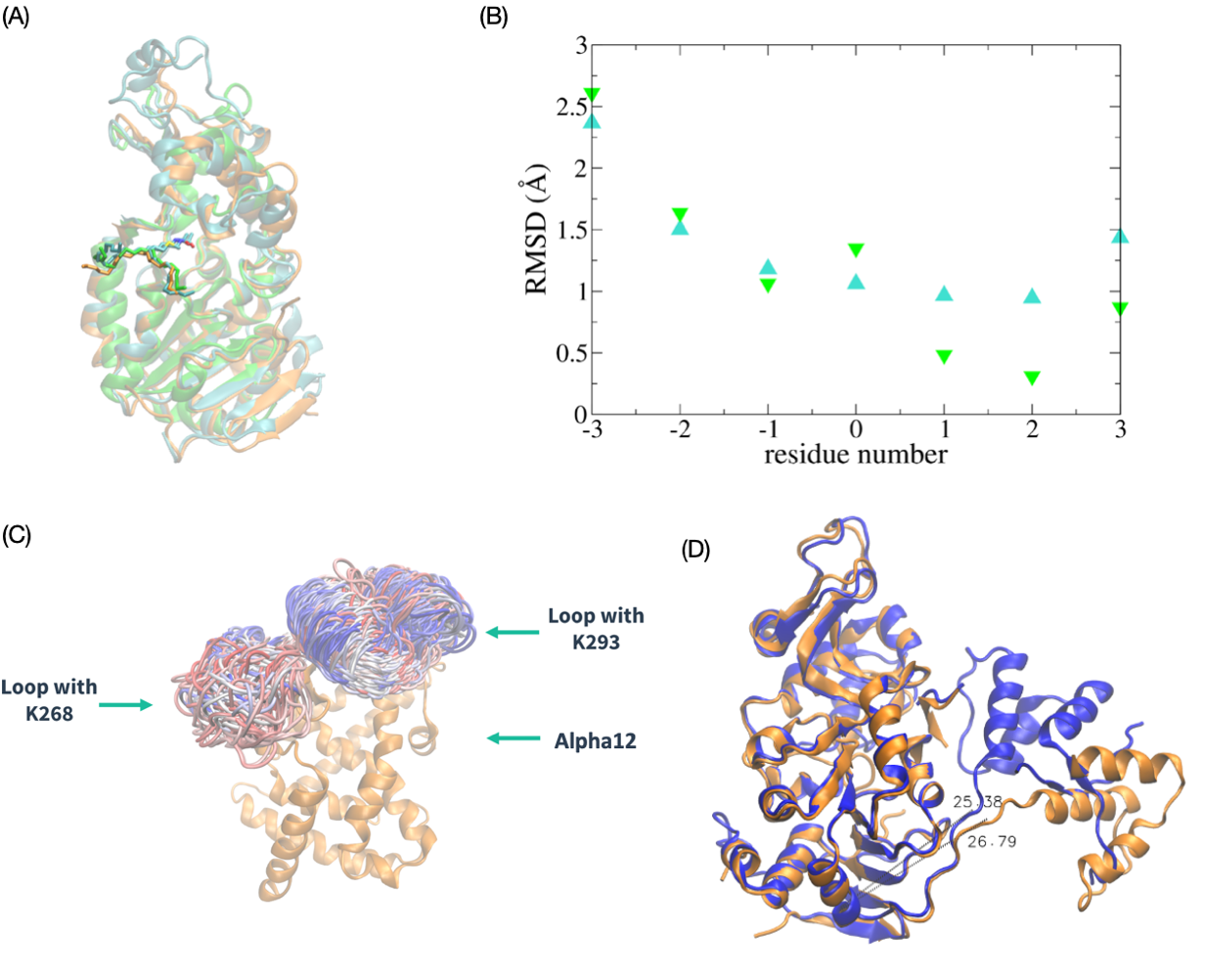


Supplementary Figure S2.  K268 and K293 peptide study for SIRT1 docking. (A) Structural alignment of the sirtuin structures, shown as cartoon with transparency for sirtuins and in licorice for the peptides. Sirtuins structural are colored as follows: orange for 4ZZJ, green for 2H4F, and cyan for 4IAO. (B) Root means square deviation of the backbone of the peptides from three sirtuin crystallographic structures (PDB ID: 4ZZJ, 2H4F [green downward triangle] and 4IAO [cyan, upward triangle]). (C) A small representative set of the 3000 configurations generated for PPARγ, showing sampling of K268- and K293-containing loops. The protein conformations along the trajectory are represented by a color gradient, ranging from red to blue, illustrating the observed structural variability. The conformations were arranged in a trajectory without being ordered amongst themselves. (D) The figure shows two structures of the human SIRT1 (blue for PDB ID: 5BTR and orange for PDB ID:4ZZJ) and the distance between the carbonylic carbon of the C-terminal of N-terminal domain and the carbonylic carbon of the N-terminal of the catalytic domain.

Supplementary Figure S3


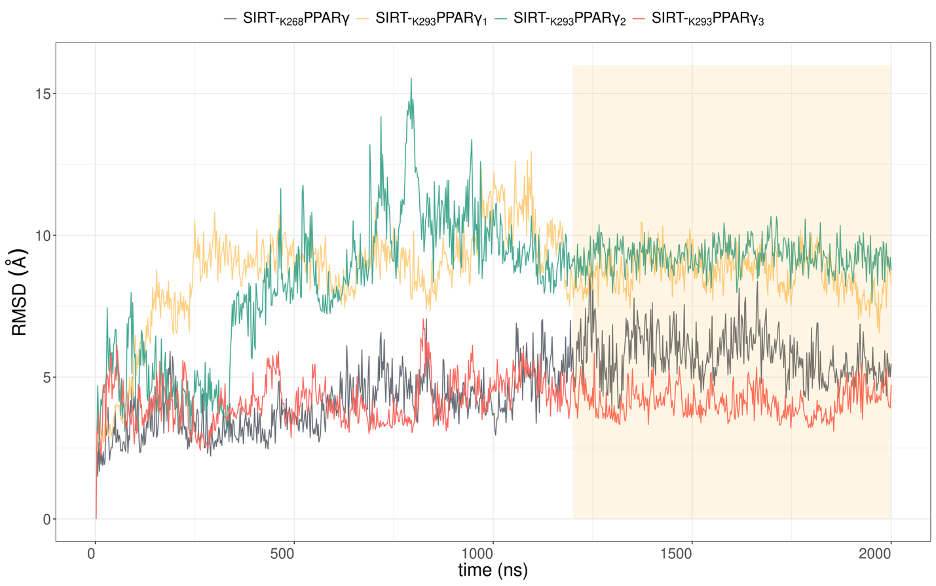
Supplementary Figure S3. RMSD along the MD simulation time for the models SIRT1-_K268_ PPARγ, SIRT1-_K293_PPARy1, SIRT1_-K293_PPARy_2_, and SIRT11-_K293_PPARy_3_.

Supplementary Figure S4.


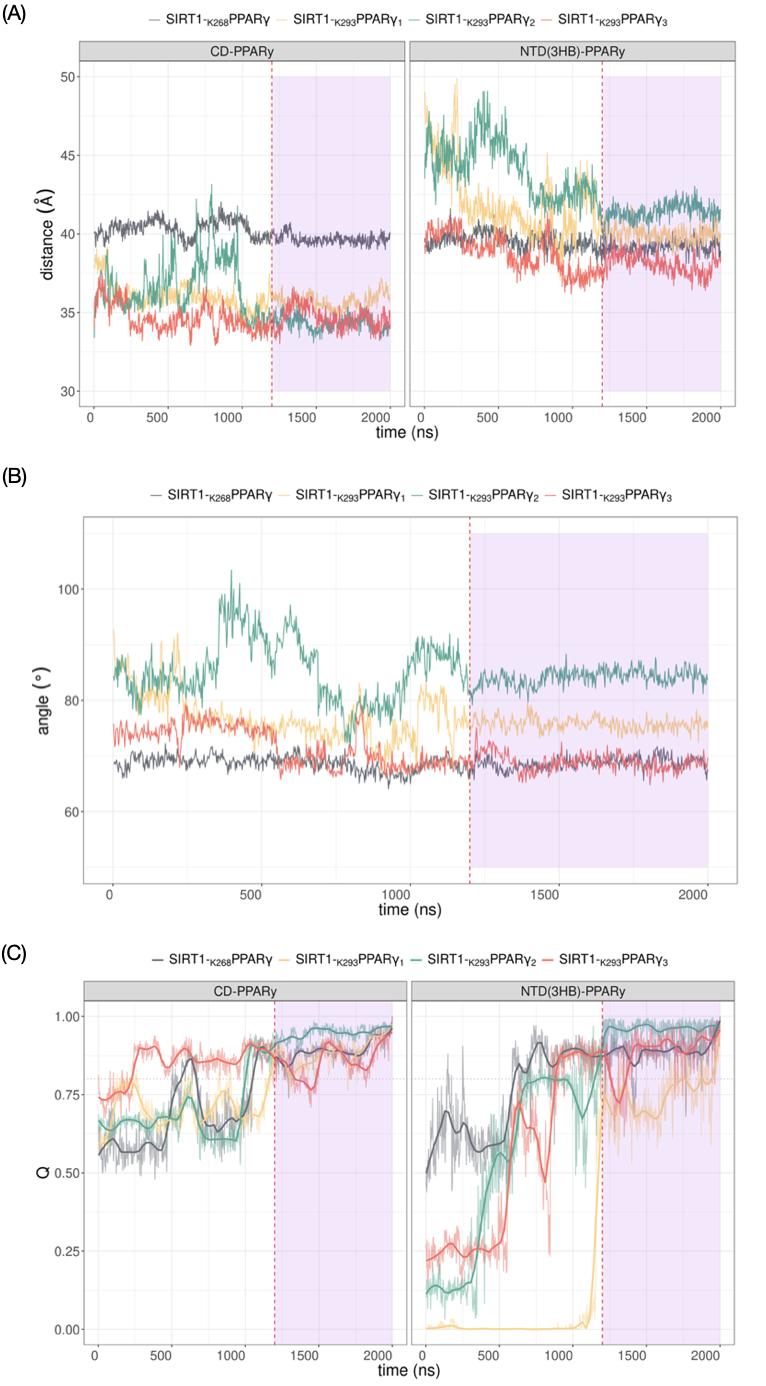


Supplementary Figure S4. Time evolution of structural parameters describing the SIRT1–PPARγ complex. (A) The plots show the distances between the geometric centers of the CD and PPARγ (left) and NTD and PPARγ (right); the left panel corresponds to the CD–PPARγ interface, and the right panel to the NTD–PPARγ interface. (B) The plot shows the angle formed by the geometric centers of the NTD, CD, and PPARγ domains (NTD–CD–PPARγ) over the course of the simulations. (C) The plots show the time evolution of the Q-score ^55^ , which quantifies the similarity of interfacial contacts between PPARγ and each SIRT1 domain, using the last frame reference. The left panel corresponds to the CD–PPARγ interface, and the right panel corresponds to the NTD–PPARγ interface. Each curve corresponds to a different model: SIRT1-_K268_-PPARγ (black), SIRT1-_K293_PPARγ_1_ (orange), SIRT1-_K293_PPARγ_2_ (green), and SIRT1-_K293_PPARγ_3_ (red). The vertical red dashed line marks 1200 ns, the point at which structural parameters converge, indicating equilibration. The shaded purple region represents the trajectory segment used for subsequent analyses.

Supplementary Figure S5.


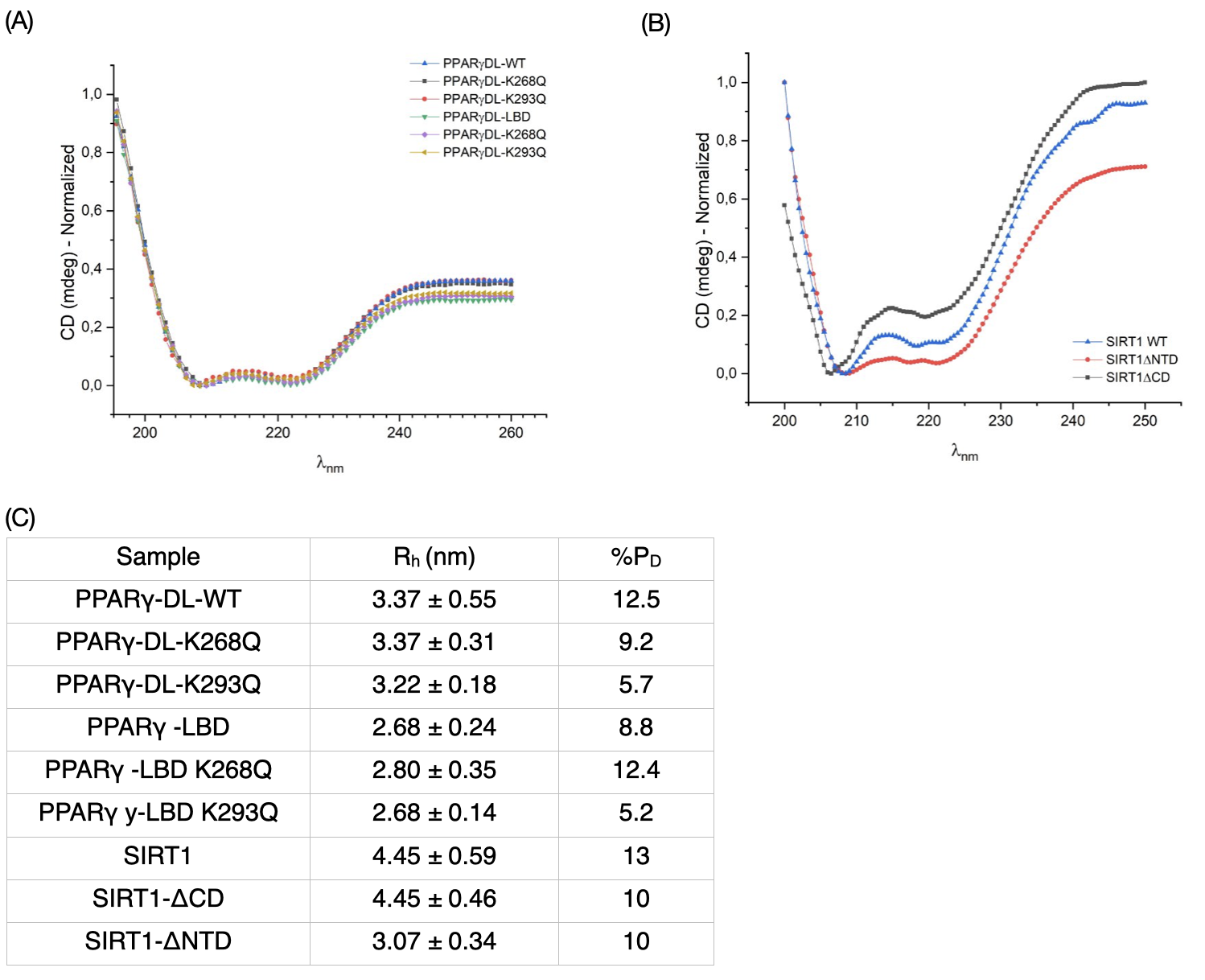


Supplementary Figure S5. Biophysical Characterization of protein constructs. (A) Circular Dichroism (CD) spectrum of   PPARγ and (B) SIRT1 constructs. (C) Table showing the Hydrodynamic Radius (R_h_) and polydispersity percentage (PD %) determined by Dynamic Light Scattering (DLS) for PPARγ and SIRT1 constructs.

Supplementary Figure S6.
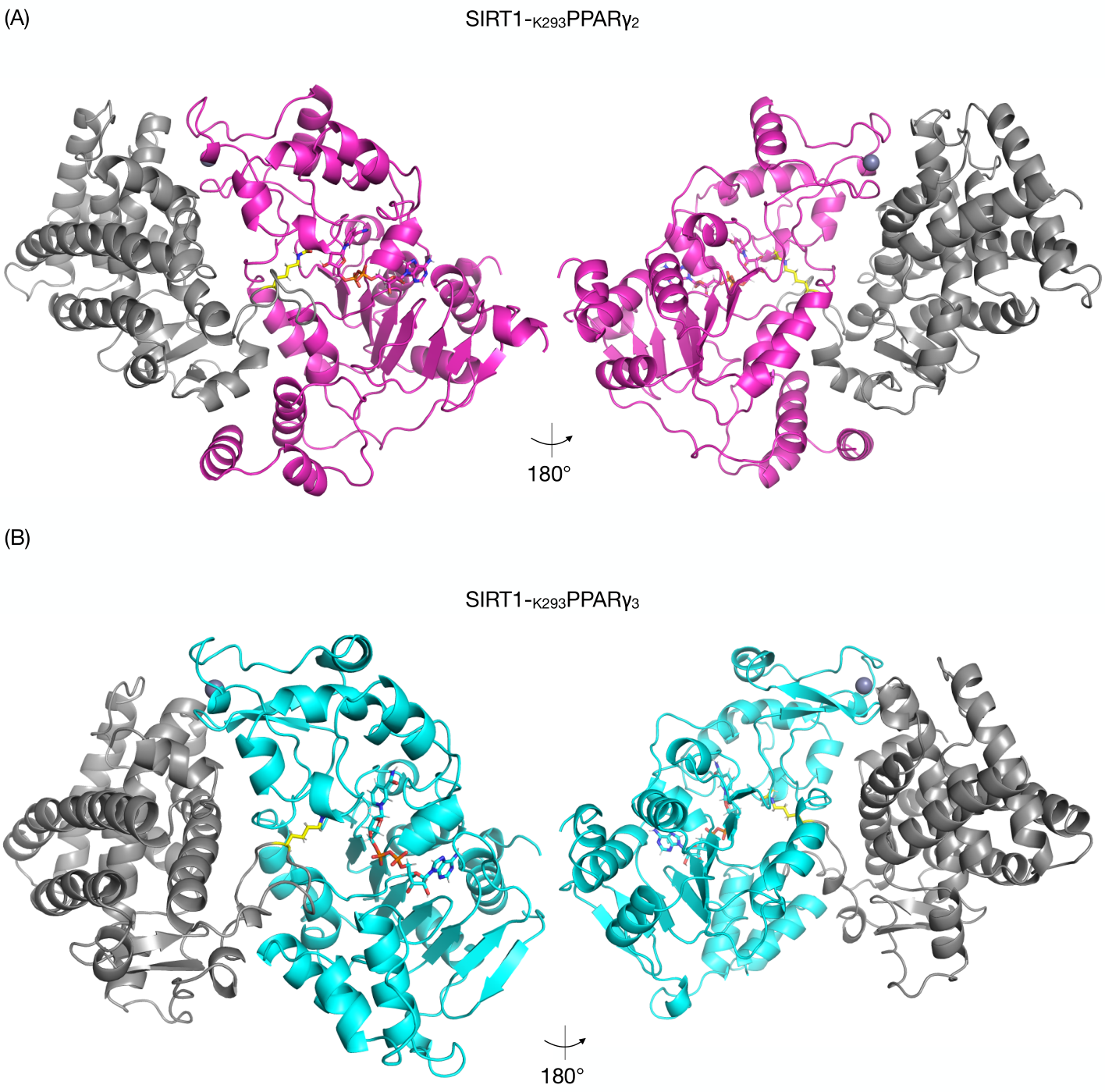


Supplementary Figure S6. Model of SIRT1-_K293_PPARγ_2_ and model of SIRT1-_K293_PPARγ_3._ PPARγ is colored gray, and SIRT1 in pink or blue and the ac-peptide side chain is highlighted in yellow.

Supplementary Figure S7.


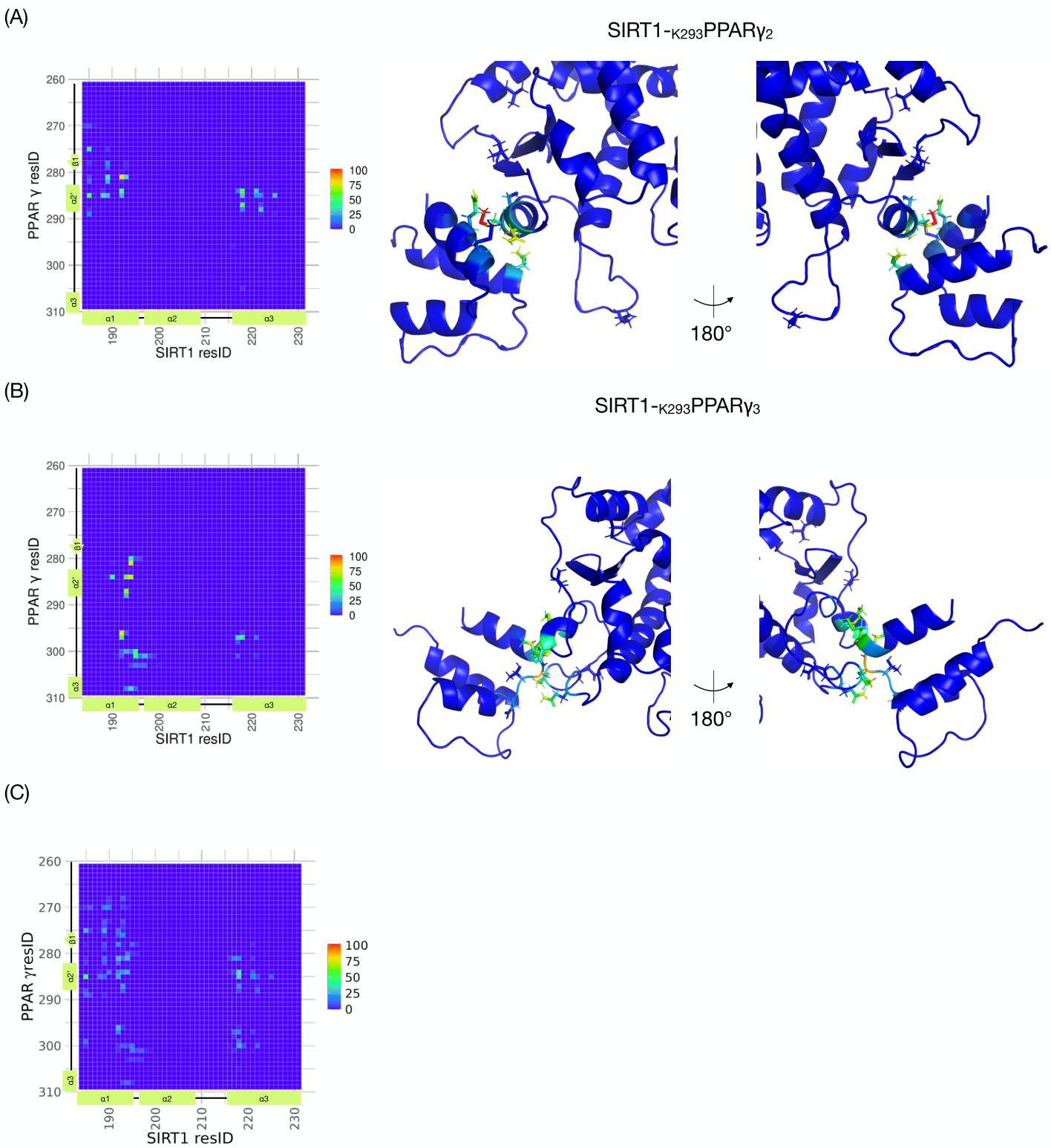


Supplementary Figure S7. Contact map from MD simulation of (A) model of SIRT1-K_293_PPARγ_2_, (B) model of SIRT1-K_293_PPARγ_3_. The contact maps represent the average distances less than 5 Å between any atom of SIRT1-NTD(3HB) and any atom of the PPARγ (LBD) over the MD trajectory. (C) Average contact map from MD simulations combining the SIRT1-K_293_PPARγ1-3 models. This represents the average interaction patterns across the last 800 ns of each of the three simulations, providing an overall view of the contact points between all models. The numbers on the y-axis and x-axis indicate the amino acid positions within the PPARγ (LBD) and SIRT1-NTD(3HB) structures, respectively. The color intensity of each cell represents the frequency of contact between the corresponding PPARγ (LBD) and SIRT1-NTD(3HB) residues.

Supplementary Figure S8.


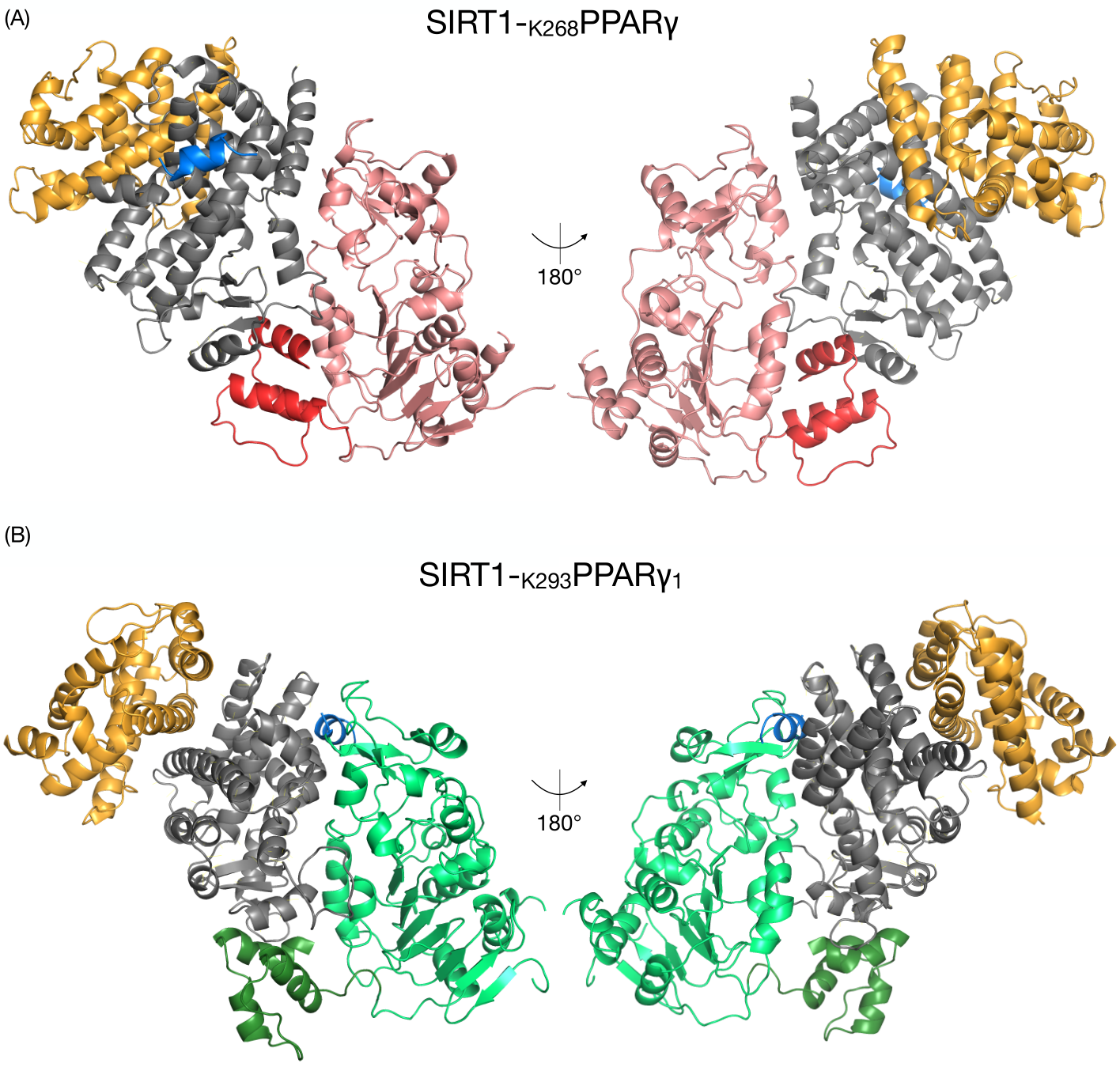


Supplementary Figure S8. SIRT1 is bound to a distinct interface of the RXR and NCoR. The superimposition of the PPARγ:RXR complex (PDB ID: 3DZY) with the SIRT1-_K268P_PARγ and SIRT1-_K293_PPARγ_1_ models illustrates the non-overlapping binding interfaces of SIRT1 compared to RXR and NCoR. The figure shows RXR (yellow), NCoR (blue), PPARγ (grey), and SIRT1 (red and green).
